# An Outdoor Aging Study to Investigate the Release of Per- And Polyfluoroalkyl Substances (PFAS) from Functional Textiles

**DOI:** 10.1021/acs.est.1c06812

**Published:** 2022-02-25

**Authors:** Steffen Schellenberger, Ioannis Liagkouridis, Raed Awad, Stuart Khan, Merle Plassmann, Gregory Peters, Jonathan P. Benskin, Ian T. Cousins

**Affiliations:** † Department of Environmental Science, Stockholm University, SE-106 91 Stockholm, Sweden; ‡ RISE Research Institutes of Sweden, Stockholm 111 21, Sweden; § IVL Swedish Environmental Institute, 114 28 Stockholm, Sweden; ∥ School of Civil and Environmental Engineering, University of New South Wales, Sydney, New South Wales 2052, Australia; ⊥ Department of Technology Management and Economics, Chalmers University of Technology, Gothenburg 412 96, Sweden

**Keywords:** PFAS, diffuse emissions, textile weathering, microplastic fibers, total fluorine analysis, functional textile

## Abstract

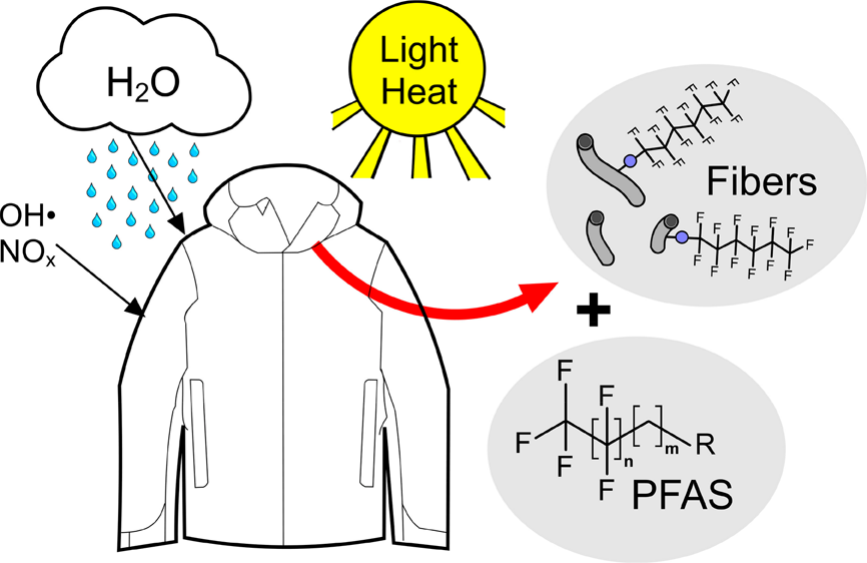

The emission of per- and polyfluoroalkyl substances (PFAS) from functional textiles was investigated via an outdoor weathering experiment in Sydney, Australia. Polyamide (PA) textile fabrics treated with different water-repellent, side-chain fluorinated polymers (SFPs) were exposed on a rooftop to multiple natural stressors, including direct sunlight, precipitation, wind, and heat for 6-months. After weathering, additional stress was applied to the fabrics through abrasion and washing. Textile characterization using a multiplatform analytical approach revealed loss of both PFAS-containing textile fragments (e.g., microfibers) as well as formation and loss of low molecular weight PFAS, both of which occurred throughout weathering. These changes were accompanied by a loss of color and water repellency of the textile. The potential formation of perfluoroalkyl acids (PFAAs) from mobile residuals was quantified by oxidative conversion of extracts from unweathered textiles. Each SFP-textile finish emitted a distinct PFAA pattern following weathering, and in some cases the concentrations exceeded regulatory limits for textiles. In addition to transformation of residual low molecular weight PFAA-precursors, release of polymeric PFAS from degradation and loss of textile fibers/particles contributed to overall PFAS emissions during weathering.

## Abstract 2

An outdoor weathering experiment demonstrated enhanced environmental losses of per- and polyfluoroalkyl substances (PFAS) resulting from various PFAS-impregnated durable water-repellent textiles.

## 1 Introduction

Functional textiles designed for outdoor activities^1^ or personal protective equipment^2,3^ are used throughout modern society. A large proportion of these products use highly fluorinated water- and stain-repellent fabric impregnations.^4−6^ While many producers of recreational outdoor textiles in the European Union have shifted to PFAS-free impregnations over the last 5–10 years due to voluntary phase-out initiatives and increasing regulatory pressure, producers of occupational textiles (e.g., in healthcare and firefighting) still use fluorinated materials to fulfill safety requirements.^7^ During use, functional textiles are subjected to considerable physical stress from sunlight, abrasion, precipitation, temperature changes, and chemicals in urban air.^8^ These stressors, in combination with laundering,^9^ may lead to the diffuse emission of chemicals into the environment, and ultimately a loss of functionality over time (e.g., loss of water droplet repellency).^10^

Fabric impregnations of modern outdoor garments and equipment are based on textile finishes often applied as durable water repellent (DWR) coatings based on side-chain fluorinated polymers (SFPs).^11^ SFPs typically consist of fluorinated alkyl side chains of different chain lengths bound to nonfluorinated polymer backbones.^12^ The fluorinated alkyl side-chain constitutes the functional unit of the polymer (see Supporting Information (SI) Figure S1) which imparts oil and water repellency to the garment. SFPs form durable polymer films on fiber materials^13^ and are designed to resist degradation from environmental stressors and washing. However, long-term weathering can lead to breakdown of the SFP and ultimately a loss of functionality. The terminal transformation products of SFP degradation, perfluoroalkyl acids (PFAAs), are among the most environmentally persistent substances known.^14^ Moreover, some PFAAs are toxic and bioaccumulative^15^ leading to their regulation^16^ as Substances of Very High Concern.^17,18^ Limit-values for textile application have been established, or proposed, for some PFAAs in Europe (e.g., 1 μg/m^2^ for perfluorooctanesulfonic acid (PFOS) (or 8.4 ppb for the PA fabrics in this study with a surface density of 119g/m^2^)^19^ and 25 ppb for perfluorohexanoic acid (PFHxA)^18^ and perfluorooctanoic acid (PFOA)^20^). A previous weathering study by van der Veen et al. demonstrated that SFP-treated fabrics exposed to ultraviolet (UV) light, heat and moisture can form PFAAs at concentrations that exceed regulatory limits.^21^ This previous study focused exclusively on the analysis of targeted PFAAs and their precursors (e.g., FTOHs) in a laboratory experiment and did not take account of the combined effects of real-world weathering. Solar radiation, heat, moisture (including salt water and acidic rain), airborne pollutants and oxidants (e.g., SO_*x*_, NO_*x*_, OH•, O_3_, soot, or dust) as well as biological factors (e.g., bird droppings) may impact polymeric textile materials in real outdoor weathering.^22^ Since simulating these conditions is challenging under laboratory conditions,^23^ certain locations with harsh climatic conditions have become accepted reference sites for large-scale aging tests.^24^ While the weathering of organic polymers including textile materials has been studied for decades to examine durability and chemical loss,^25^ little is known about the related loss processes specifically for functional textiles with SFP treatments.

Given these knowledge gaps, this study aimed to assess the breakdown of SFP-coated textiles weathered under real-world outdoor conditions. Fabrics treated with different SFPs typically used for high-performance water- and stain-repellent textiles were exposed to ambient conditions during spring and summer (for 3 and 6 months) on a rooftop in Sydney, Australia. After weathering, the textiles were subjected to further abrasion and washing to mimic additional stress applied during use.^26^ Thereafter, the textiles were assessed for morphological and chemical changes using a multiplatform analytical approach. To the best of our knowledge, this is the first study to investigate losses of both low molecular weight PFAS and microfibers coated with SFPs under real-world conditions. The results were used to explain relevant loss processes of outdoor textiles and their contribution to PFAA emissions under real-world conditions.

## 2 Materials and Methods

### 2.1 Experimental Approach

**Figure 1:**
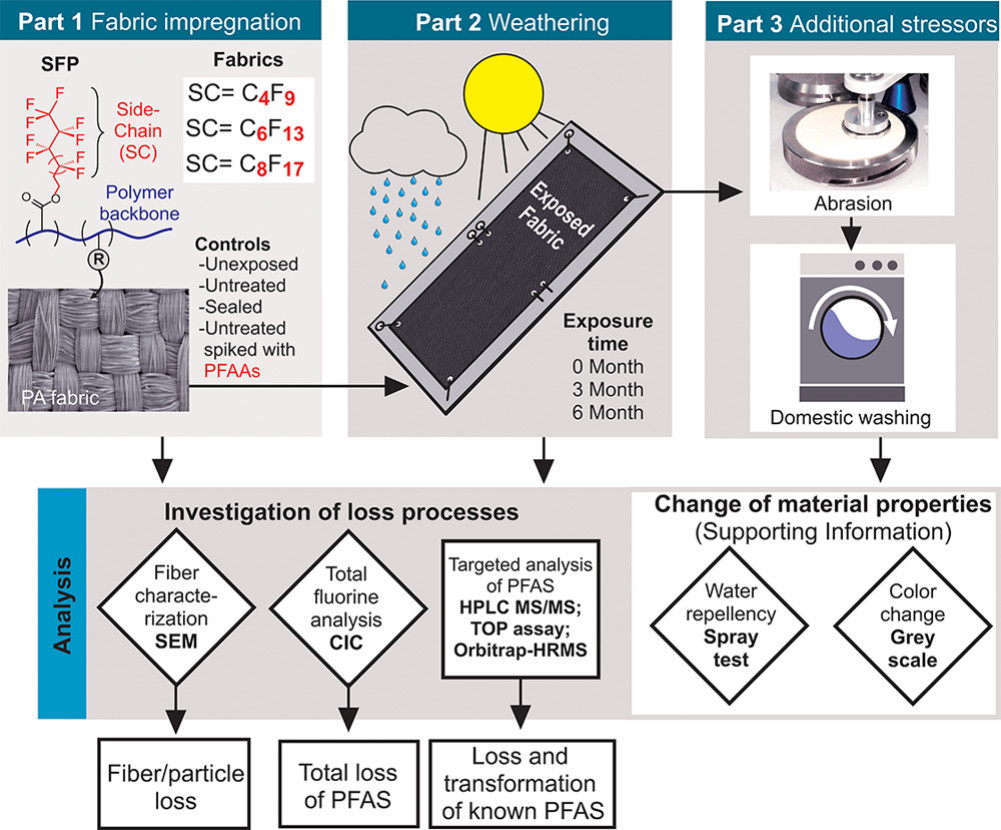
Overview of the experimental setup, consisting of fabric treatment (Part 1), outdoor exposure of the textile fabrics (Part 2), and application of abrasion and washing (Part 3). All three experiments were subjected to comprehensive characterization including scanning electron microscopy (SEM), combustion ion chromatography (CIC), high performance liquid chromatography-tandem mass spectrometry or high-resolution mass spectrometry (HPLC-MS/MS or HPLC-HRMS), and the total oxidizable precursor (TOP) assay. Additional changes of material properties were examined by measuring the water repellency (method: spray test) and the color change (method: gray scale).

The experiment consisted of 3 parts (Figure 1). Part 1 involved a wet chemical treatment process for impregnation of polyamide (PA) fabrics with SFPs based on different side-chain moieties (C_4_F_9_-R, C_6_F_13_-R, and C_8_F_17_-R; SI Figure S1). The impregnated textiles were then subjected to a static, real-time weathering experiment (involving *n* = 2 replicates per fabric type; Part 2), and thereafter, mechanical abrasion and washing steps (Part 3) to simulate additional stress parameters during use. Characterization of fabrics involved determination of fiber surface defects by scanning electron microscopy (SEM; Parts 1–3), determination of fluorine content by combustion ion chromatography (CIC; Parts 1–3), and targeted PFAS analysis by liquid chromatography tandem mass spectrometry (HPLC-MS/MS; Parts 1–3) for nonvolatile PFAS. The total oxidizable precursor (TOP) assay^27^ and targeted analysis of fluorotelomer alcohols (FTOHs) was performed on textile extracts from Part 1 after fabric treatment. Finally, the impact of weathering-induced changes in textile morphology and chemistry on material performance were determined by measuring the textiles’ water repellency (spray test; ISO 4920^13^) and color fading (gray scale test; ISO 105 A02:1995^28^) after Parts 1–3 (see Figure S5, Table S8 and Table S9 in the SI). Standards and reagents used for all parts of the study are listed under SI Table S1.

#### 2.1.1 Fabric Preparation

An untreated (SFP-free) PA fabric (polyamide 6,6 made from hexamethylenediamine and adipic acid monomers each containing six carbons) with durable rip-stop pattern and 115 ± 5 g/m^2^ (60 ± 1 threads per cm warp and 33 ± 1 threads per cm weft) fabric surface density (FOV AB Sweden) was used in the in-house pad-dry-cure finishing process,^9,14^ which applied drying and chemical cross-linking to the DWR formulation. These formulations are commercially relevant water-based SFP emulsions with different side-chain modifications (C_4_F_9_–, C_6_F_13_–, and C_8_F_17_−) and other chemical ingredients. The formulations were kindly supplied by major raw material manufacturers to prepare water-based DWR formulations. The close collaboration with these raw material suppliers facilitated application of the DWR-polymers at the laboratory scale in a manner that was consistent with conditions used in the textile producing industry. The DWR formulations chosen for this study underwent performance and durability tests which were documented in a previous study.^8^ Further information on the formulations and their method of application is provided in SI Table S2.

#### 2.1.2 Weathering Experiment

SFP-treated fabrics and controls were exposed on a rooftop in Sydney, Australia ((33°54′26.8″S151°14′16.9″E)), for periods of three months (August 27 to November 26, 2017, “spring”) and six months (November 27 2017 to February 25, 2018, “spring and summer”). The test location was chosen because of its high average monthly irradiation (644 MJ/m^2^, rate for the year), which is comparable to international reference locations for outdoor textile weathering (e.g., 550 MJ/m^2^ for Florida;^24,29^ see SI Tables S3 and S4 for a comparison of average meteorological data). Over the course of the experiment, cumulative rainfall was 151.4 mm (spring) and 127.8 mm (summer), respectively, while the average temperature was 14.2 °C (range 6.5–37.3 °C) and 19.9 °C (range 14.9–43.7 °C), respectively. The high exposure to UV light and hot roof temperatures in the weathering experiment, with a long (albeit arbitrary) duration of 6 months, can be considered a worst-case user scenario for functional textiles. Further details of the weathering experiment are provided in SI Tables S3–S5. Fabrics were fixed on a custom-built stainless-steel (3 mm) modular fabric holder (SI Figure S3). Each fabric treated in a batch (35 cm × 40 cm) was divided into two parts (SI Figure S4). One part served as unexposed control, while the other part was fixed with eyelets (stainless steel, 11 mm; Prym; Germany) and cable ties (92 mm Ty-rap, stable toward UV light) to the holders and underwent weathering on the roof. Fabrics were attached to the holder so that they hung freely at ∼2.5 cm distance above the roof material.

#### 2.1.3 Abrasion and Washing

After weathering, abrasion was applied to the fabrics (Martindale abrasion;^30^ ISO 12947–2; Martindale 3000 rubs with 9 Pa) followed by one domestic washing cycle^37^ (ISO 26330 ISO, 2001, 40 °C) to remove loose particles and fiber fragments (details in SI Table S6).

#### 2.1.4 Characterization of Textile Surface Defects

Scanning electron microscopy (SEM) was used to investigate the impact of weathering, washing, and abrasion on the textile weave and fibers. The different fabrics were systematically analyzed with SEM overview pictures (low magnifications) and for surface defects on a fiber level by analyzing weft and warp yarns with high magnification (SI Table S7).

#### 2.1.5 Total Fluorine Determination

Total fluorine (TF) analysis was carried out by CIC using an AQF-2100H combustion unit (Mitsubishi, Japan) which was coupled to a Dionex ICS-2100 Integrion IC (Thermo Scientific, U.S.) described in more detail by Schultes et al.^31^ Subsampling for TF analysis involved collection of small fabric pieces (∼0.5 mg) before and after weathering (*n* = 4 samples of each textile), washing (*n* = 4 samples of each textile), and abrasion (*n* = 4 samples of each textile). Since the fabrics were treated in a noncontinuous batch process, some inter- and intratextile variability in the DWR finish was expected. For each textile, a stencil was used to cut out pieces to match the sampling location of exposed and unexposed fabrics (*A* = 2 cm^2^; SI Figure S4). In addition, the homogeneity of fluorine content on one fabric (C_8_F_17_–SFP coated) was assessed via TF determination on random samples from different locations (*n* = 9) on the same fabric (SI Figure S13). Further details of the TF analysis are provided in S1.1 of the SI.

#### 2.1.6 Targeted PFAS Analysis

Textile samples of approximately 1 cm^2^ were weighed and extracted with methanol and analyzed using a Waters Acquity ultraperformance liquid chromatograph (UPLC) coupled to a Waters Xevo TQS tandem mass spectrometer (MS/MS) for 48 different PFAS (SI Table S1). A detailed method is also provided in the SI under S1.2.

In order to assess the total quantity of PFAA-precursor residuals, each textile (1 cm^2^; pre-weathering) was extracted with methanol and the extracts were subjected to a modified version of the TOP assay^27^ as described by Liagkouridis et al.^32^ The goal was to remove low molecular weight PFAS, while leaving SFPs on the fabrics, as shown by other studies using this procedure.^33−35^ Application of the TOP assay transformed the extractable PFAA-precursors to PFAAs, which were analyzed by HPLC-MS/MS. Further details are provided in S1.3 in the SI. Textile extracts of unexposed (non-weathered) fabrics with C_8_F_17_ SFP treatment were also analyzed for 8:2 and 10:2 fluorotelomer alcohols (FTOHs) using a Dionex UltiMate 3000 ultrahigh performance liquid chromatograph coupled to a Q Exactive HF Orbitrap high-resolution mass spectrometer (Thermo Scientific). A detailed method description can be found in S1.4 in the SI.

#### 2.1.7 Quality Control

A total of five different controls were included in this study in order to pinpoint the source of chemical loss in the weathered fabrics, and rule out losses from, for example, shipping and handling of samples (see SI Figure S3). “Stay controls” consisted of *n* = 2 replicates of each type of fabric (untreated and C_4_F_9_–, C_6_F_13_–, and C_8_F_17_–SFP treatments) which were sealed and stored in the dark at room temperature in Stockholm for the duration of the experiment. “Shipping controls” consisted of *n* = 2 replicates of each of the four fabric types which were sealed and shipped to Australia, and then stored in the dark at room temperature for the duration of the experiment, and then shipped back to Stockholm. “Untreated controls” consisted of *n* = 2 replicates of the PA fabric, which were not treated with SFP or fortified with PFAAs. These controls were exposed alongside coated fabrics in order to measure contamination potentially introduced from the sampling site (e.g., via rain and/or particulate and/or gas-phase PFAS that may sorb to the fabric). “Spiked controls” (*n* = 3) consisted of a portion of an untreated fabric spiked with aqueous solutions of PFOA, PFHxA, and PFBS (PFAAs which are not expected to degrade) and marked with position tags. These controls were exposed alongside coated fabrics in order to assess losses incurred during the experiment, for example by precipitation. Finally, “sealed controls” consisted of *n* = 2 replicates of each treated fabric which were sealed in polypropylene plastic bags, covered with opaque tape, and then placed on the rooftop where they were sampled together with real samples at 3 and 6 months. The purpose of these controls was to quantify losses associated with heat. All controls were prepared and analyzed at the same time by SEM microscopy (surface defects), UPLC-MS/MS (targeted PFAS analysis) and CIC (total fluorine).

#### 2.1.8 Data Handling and Data Analysis

Target PFAS concentrations were converted to fluorine equivalent concentrations (*C*_F_PFAS_, ng F/g) according to eq 1:
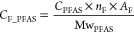
1where *C*_PFAS_ (ng PFAS/g) and *n*_F_ (mol) are the concentration and number of fluorine atoms for a given target, respectively, AF is the molar mass of fluorine (18.998 g/mol), and Mw_PFAS_ (g PFAS/mol) is the molecular weight of the target. Once the concentrations were converted to fluorine equivalents, they were summed to obtain ∑*C*_F_PFAS_ concentrations, which were directly comparable to TF measurements. In cases where a target was below detection limits, the concentration was replaced with a value of “0” for determining sum concentrations.

Fabric shrinking is a common effect after textile outdoor exposure, which can result in a change in surface area.^36^ Consequently, TF losses are expressed on a weight basis [in μg/g] to avoid confounding observations from changes in surface area (see SI Figure S17 and S2.2 for measurements and a detailed discussion).

#### 2.1.9 Statistical Analysis

Statistical tests (in Microsoft Excel) were used to evaluate the variation in measurements and significance of all observed changes. In all cases a significance level (α) of 0.05 was used. Outliers that exceeded the interquartile range (IQR) were not included in calculation of means (additional details are provided in S1.6 in the SI).

## 3 Results and Discussion

### 3.1 Textile Characterization Preweathering

**Figure 2:**
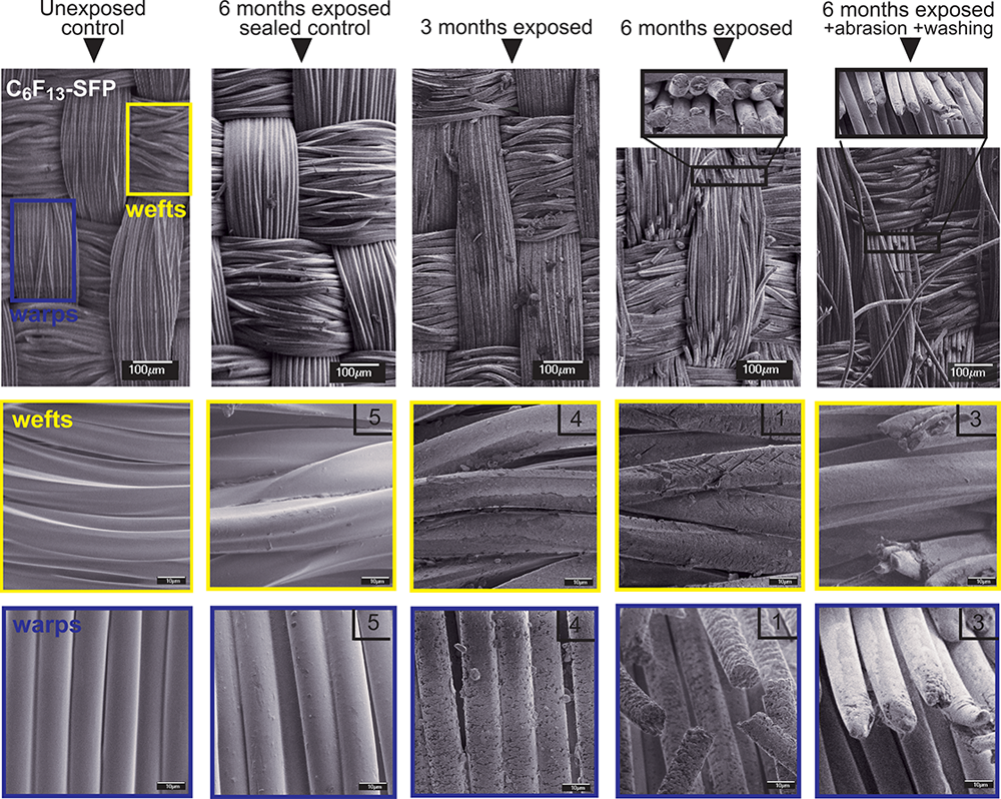
SEM pictures of a PA rip-stop fabrics treated with a C_6_F_13_–SFP finish. Unexposed controls are shown in comparison to “sealed controls” and exposed fabrics with different exposure times and after applying abrasion and washing. Warps (blue frames) and weft fibers (yellow frames) are also displayed in higher magnification. Numbers in the upper right corner of the SEM pictures refer to the color change assessed with the Gray scale in comparison to the unexposed fabrics (scale 1–5; 5 = no visual change 1 = a large visual change).

Prior to weathering, TF concentrations were highest in the C_4_F_9_-treated textile (4730–5130 μg/g) followed by C_8_F_13_- (4280–5060 μg/g) and C_6_F_13_– (4540–4700 μg/g). These concentrations equate to 0.60 g F/m^2^ (C_4_F_9_), 0.56 g F/m^2^ (C_8_F_17_) and 0.54 g F/m^2^ (C_6_F_13_) calculated on surface area basis, assuming a fabric weight of ∼119 g/m^2^. Comparable values ranging from 0.02 to 0.7 g F/m^2^ in commercial textiles were reported previously.^34,37,38,32^ Replicate (*n* = 9) analyses of TF on a C_8_F_13_-treated textile revealed low relative standard deviation (<4%; see SI Figure S12), indicating a homogeneous distribution of SFPs over the fabric (confirmed by SEM microscopy; Figure 2). Water repellency was tested before outdoor exposure and showed high spray ratings for all fabric treatments (SI Figure S16) which confirmed the materials’ functionality and a homogeneous distribution of the DWR finish.

### 3.2 Textile Characterization Postweathering

TF concentrations were not statistically different between “shipping” and “stay” controls for all SFP treatments, indicating no loss of fluorine during shipping (Figure 2). TF concentrations were also consistent between 3- and 6-month “sealed” controls (all treatments), but these concentrations were significantly lower than “shipping” and “stay” controls for the C_4_F_9_-and C_8_F_17_–SFP coated textiles (see ANOVA statistics in SI Tables S16–S18), possibly due to heat-induced off-gassing of volatile low molecular weight PFAS in the first 3-months. Although no fiber surface defects were visible for the sealed samples, some cracks in the SFP coating were visible in between the fibers (see, e.g., the 6-month sealed sample in Figure 2). Nevertheless, these results generally point to minimal changes to the fabrics in the absence of weathering or during shipping and handling of samples.

**Figure 3:**
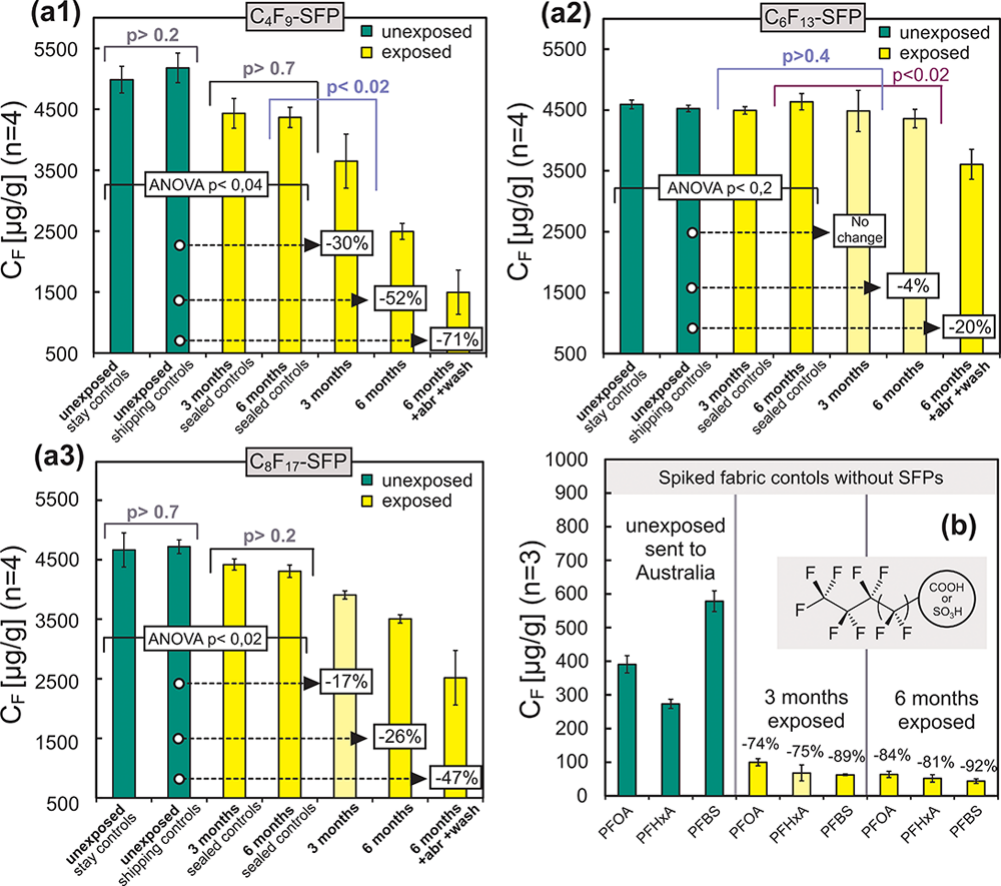
Total fluorine content of fabrics before and after weathering and after the additional abrasion test and washing. PA fabrics measured with CIC contained different fluorinated textile finishes based on “short-chain” SFPs with (a1) C_4_F_8_ and (a2) C_6_F_13_ side chains as well as (a3) “long-chain” SFPs based on C_8_F_17_ side chains. (b) Shows untreated fabrics that were spiked with PFAAs and underwent weathering as well.

For exposed fabrics, a statistically significant decrease in TF concentrations (relative to controls) with exposure time was observed (Figure 3) which accompanied severe deterioration of the textile fibers (Figure 2). After 6 months, TF concentrations were reduced by 4, 26, and 52% for C_6_F_13_, C_8_F_17_, and C_4_F_9_-based SFPs, respectively, relative to unexposed controls. Major surface defects were also observable including breaking and loss of fibers from both warp and weft yarns, and degradation of individual fiber cores (Figure 2). Further TF loss and fiber degradation was observed following abrasion and washing of the 6-month weathered textiles. TF levels were reduced by 20, 47, and 71%, for C_6_F_13_, C_8_F_17_, and C_4_F_9_-based SFPs, respectively, after weathering, abrasion, and washing, relative to unexposed controls. (Figure 2 and SI Figures S7 and S8). Fibers were also disoriented and more loosely joined in the weave, while warp edges were more rounded at the ends after abrasion and washing (Figure 2 and SI Figures S7 and S8). This indicated loss of not only larger fiber fragments, but also smaller particles from abrasion and washing. Black PA fabrics (original color provided by the fabric manufacturer) faded to gray after weathering, possibly due to an increase in fiber surface defects which increases the diffuse light reflection, resulting in a lighter appearance (SI Figure S9). However, the loss of discolored surface fibers during abrasion and washing resulted in a darker fabric color due to revealing of unexposed fibers (black) from below (SI Figure S10). In addition, the water repellency of fabrics was reduced for all the exposed fabric SFPs (see SI Figure S16).

### 3.3 Targeted Analysis of PFAAs

**Figure 4:**
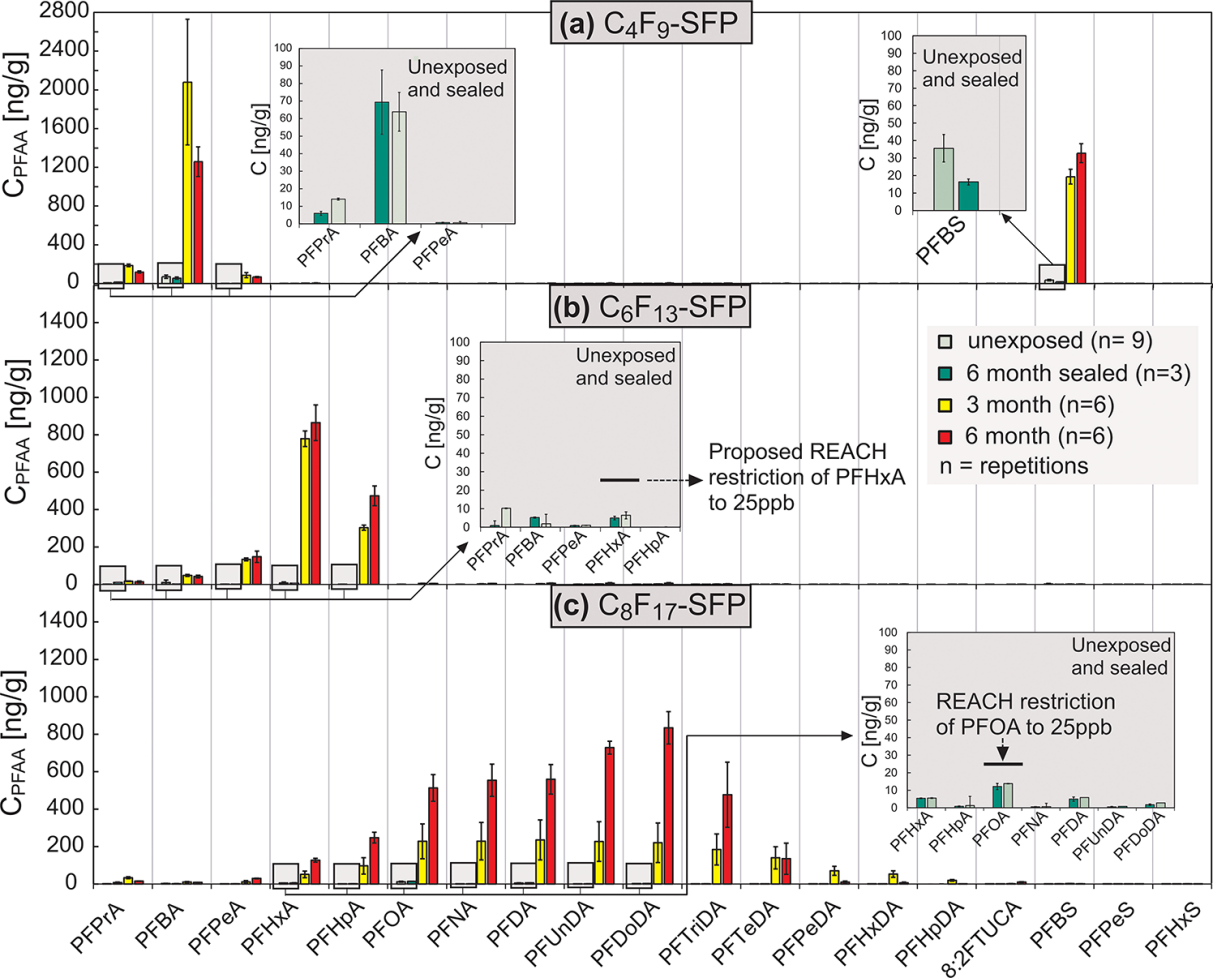
Summary of the targeted analysis of PFAAs after weathering of textiles with (a) short-chain C_4_F_9_– and (b) C_6_F_13_–SFPs as well as (c) long-chain C_8_F_17_–SFPs.

In spiked controls, between 74% and 92% of the PFOA, PFHxA, and PFBS added to the textiles was lost after 6-months of weathering (Figure 3b), relative to unexposed controls, indicating that water-soluble PFAAs were removed (likely by rain) during the experiment. Thus, measured PFAA concentrations in exposed SFP-coated textiles are likely large underestimates of the total PFAAs lost during the experiment. Despite these expected losses, all three SFP treatments showed a large (up to 100-fold) increase in PFAA concentrations after weathering (Figure 4), relative to unexposed controls. This trend has also been observed by van der Veen et al. in a study with commercial textiles where PFAA concentrations increased by 5- to more than 100-fold after weathering.^21^

In contrast, sealed, stay, and shipping controls all displayed low and consistent PFAA concentrations, indicating that sample handling only had a minor impact on measured PFAA concentrations. Moreover, PFAAs were not observed on untreated textile fabrics after 6 months of exposure (SI Figure S15 and Table S19), demonstrating that uptake of PFAS from the surrounding environment was negligible during the experiment (e.g., from air, dust, or precipitation). Collectively, these results demonstrate that the occurrence of PFAAs from the SFP coated textiles was solely due to weathering on the roof top.

PFAA profiles generated from weathering were unique and highly dependent on the DWR coating. While weathering of C_8_F_17_–SFP produced a range of short- and long-chain PFAAs (with perfluoroalkyl chain length of 3–15 carbons; Figure 4c), C_4_F_9_ produced almost exclusively perfluorobutanoic acid (PFBA) and perfluorobutanesulfonic acid (PFBS), while C_6_F_13_–SFP produced mostly perfluorohexanoic acid (PFHxA) and perfluoroheptanoic acid (PFHpA). The absence of long-chain PFAAs for fabrics treated with “short-chain” DWRs tested in this study reflects changes in production by chemical manufacturers in order to comply with the current regulation of PFOA^20^ and other long-chain PFAAs.^39^

The targeted analysis also revealed that fabrics with initially low PFAA concentrations can exceed regulatory levels after weathering. For the long-chain C_8_F_17_–SFPs, the concentration of PFOA increased from 12 ± 3 ng/g (ppb) for the unexposed sample to 228 ± 93 ng/g (ppb) after 3 months and to 513 ± 71 ng/g (ppb) after 6 months of weathering. Thus, for the 6-month sample the concentration was 20-fold higher than the regulatory limit value of 25 ppb in textile products.^20^ The observed change was even higher for the other long-chain PFCAs such as perfluorododecanoic acid (PFDoDA), which increased from 2 ± 0.8 ppb to 834 ± 87 ppb after 6 months. Considering the new proposal for the regulation of PFHxA in the European Union,^18^ (i.e., 25 ppb in articles), the C_6_F_13_ “short-chain” SFPs would also breach the proposed guideline after weathering. PFHxA was detected at a concentration of 779 ± 42 ppb after 3 months of exposure and at 865 ± 95 ppb after 6 months. In milder conditions associated with consumer usage these high concentrations might not be reached; nevertheless, concentrations in excess of the regulatory limits are still plausible.

**Figure 5:**
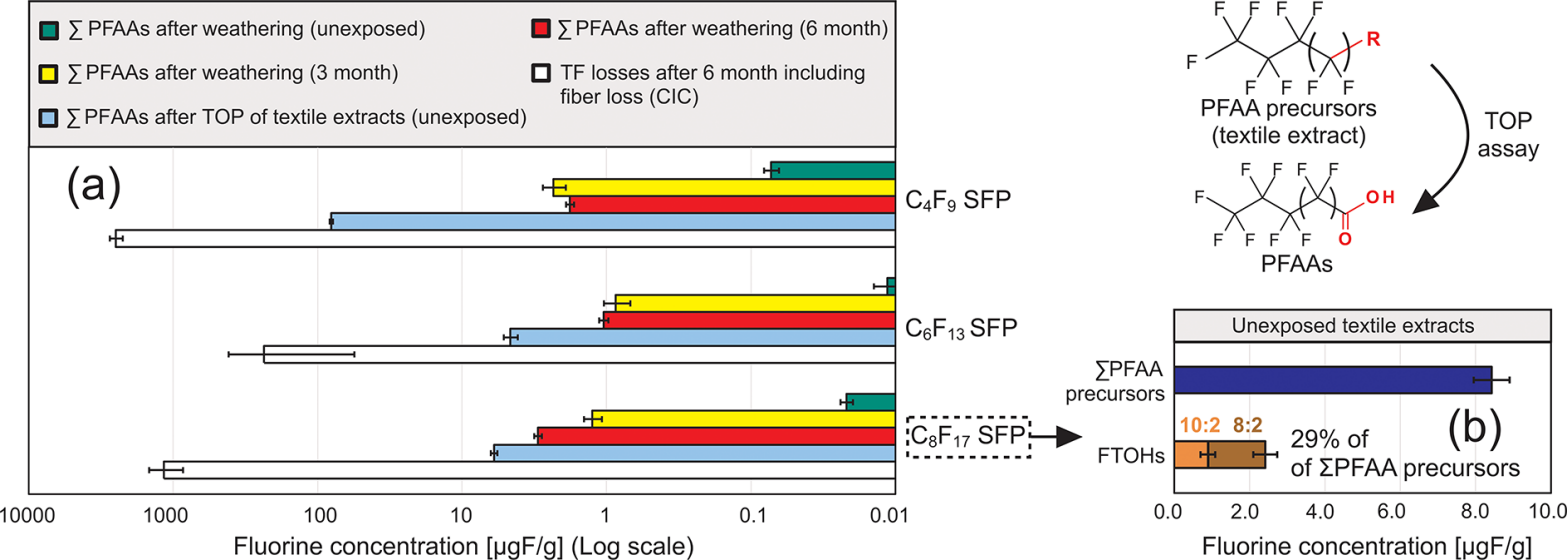
In (a) the sum of PFAAs detected after roof-top weathering were compared to the sum of PFAAs detected after extraction of unexposed fabric and application of a TOP assay (on the basis of the fluorine content of the PFAAs). The sum of PFAAs were also compared to the total fluorine losses detected with CIC (log scale). (b) Compares targeted FTOHs in textile extracts of unexposed fabrics with the sum of PFAA precursors.

In Figure 5 the analysis of targeted PFAAs, FTOHs and CIC measurements are displayed based on total fluorine concentration. During the weathering experiment a fraction of PFAAs formed were lost due rain and also due to fiber loss (when PFAAs were bound to the fiber surface). Thus, the TF concentrations in Figure 5 are not directly comparable on a mass balance basis. Nevertheless, the fluorine concentrations for all PFAAs found in the textile fabrics after 3- and 6-months of weathering were (despite losses), ∼12 to ∼270 times higher than in unexposed fabrics, which can only be attributed to precursor transformation during outdoor exposure.

Figure 5b shows the fluorine content associated with all extractable PFAA precursors in unexposed C8-SFP treated fabrics. A comparison with unexposed C8-SFP fabrics without using the TOP assay after extraction (Figure 5a) revealed a ∼60 to ∼7300-fold increase in fluorine concentrations for oxidized samples. This suggests that there is a large quantity of extractable PFAA precursors present in SFP-treated fabrics prior to weathering. Several studies have applied the TOP assay to commercial textiles. By comparing the sum extractable PFAAs before and after oxidation these studies also reported higher PFAA levels after application of the TOP assay, ranging from 10- to 860-fold higher PFAA levels.^37,34,35^

Figure 5b also shows that only 29% of the total PFAA precursors can be attributed to FTOHs, indicating that a large quantity of unidentified extractable precursors occur in these samples. We should note that the estimations in Figure 5b are based on the assumption that FTOHs were completely lost during the solvent evaporation step (prior to oxidation), and therefore did not contribute to the observed PFAA profiles following application of the TOP assay.^34^ Total PFAA precursors are then calculated as the sum of FTOHs and PFAAs post-TOP.

Under the assumption that only mobile nonpolymeric PFAS were captured by textile extraction, the sum of oxidizable precursors in unexposed C8-SFP fabrics (Figure 5b) represents the maximum amount of PFAAs that could be formed from residuals during weathering (and other processes such as washing). By comparing this maximum amount of mobile PFAA precursors in nonweathered samples with the total fluorine loss measured by CIC after 6 months of outdoor exposure (Figure 5a) the fluorine concentration of mobile precursors was still ∼140 times lower than the losses measured by CIC. Since the CIC measurements also account for particle- and fiber-loss during weathering (Figure 2) this comparison indicates that mobile residuals might have a much lower contribution to the total PFAS emissions than losses associated fibers and particles (where a large fraction of PFAS occurs as SFP).

### 3.4 Implications for Chemical Loss Processes

A TF or PFAS mass balance is impossible in this experiment because the loss of TF or PFAS due to wash-off from rain or volatilization was not quantified. The stability of SFPs is debated^40−43^ and the present study could not prove definitively that photooxidation led to cleavage of the fluorinated side chains from the polymeric backbone and ultimately PFAA formation. Therefore, PFAAs observed in the textiles after weathering could be formed due to either oxidation of low molecular weight PFAA-precursors present as textile residuals or through cleavage of fluorinated side chains from the polymers themselves.

**Figure 6:**
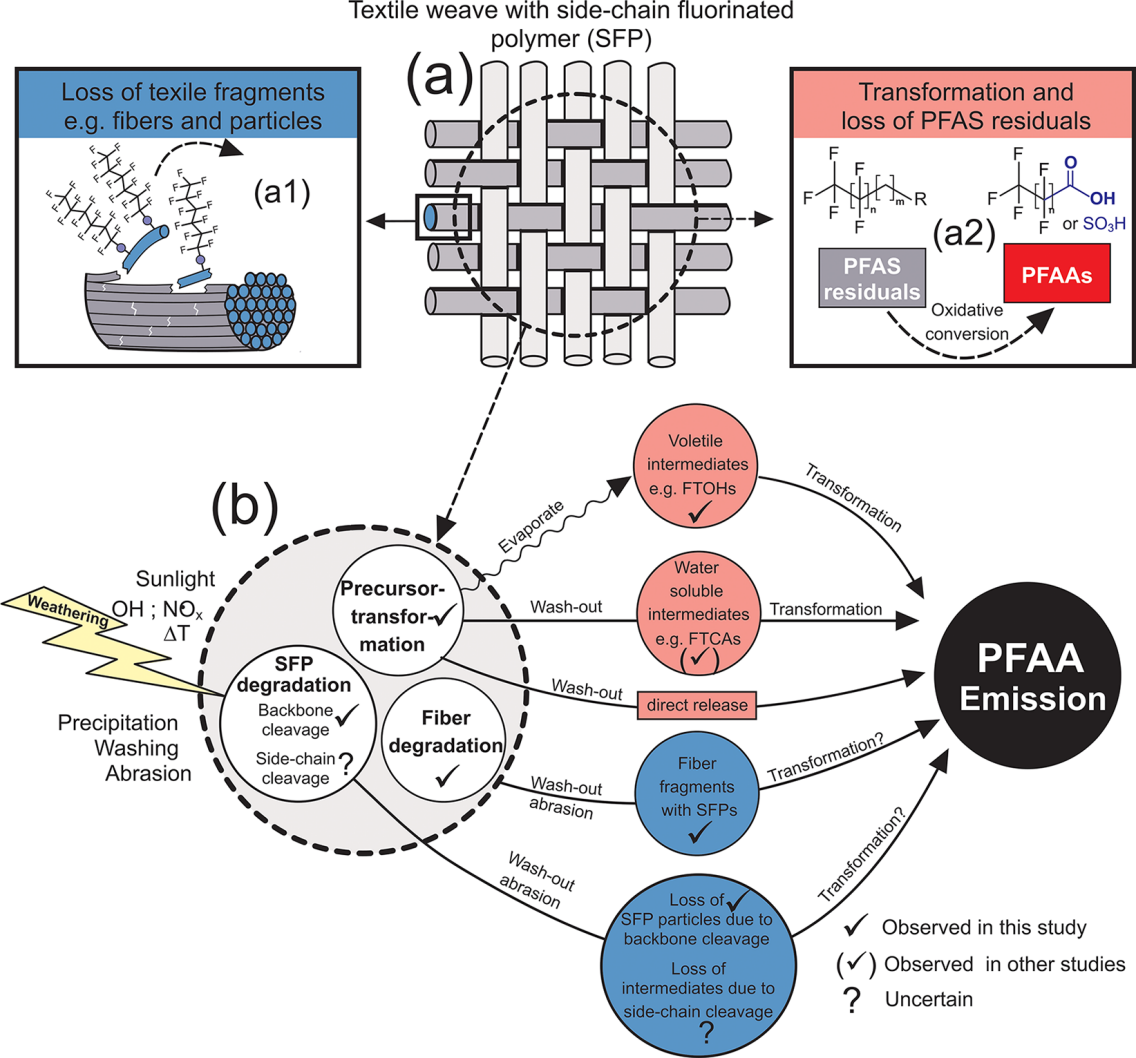
Schematic representation of loss mechanisms that that are likely to occur to (a) textiles with SFP finishes during weathering: (a1) Loss of larger textile fragments such as fibers and particles and (a2) the oxidative conversion of PFAS impurities. (b) Displays further a simplified summary of emission pathways that lead to emission and accumulation of PFAAs in the environment.

There are therefore several potential emission pathways for PFAS in the present study (Figure 6): (i) loss of larger textile fragments such as fibers and particles (Figure 6a1); (ii) degradation of the SFP from the textile fibers due to backbone cleavage (without side-chain cleavage); (iii) oxidative conversion and cleavage of fluorinated side chains of SFPs and loss of low molecular weight PFAS; and (iv) oxidative conversion and loss of mobile low molecular weight PFAS impurities (Figure 6 a2). These processes are likely to occur simultaneously and cannot be easily separated from one another. During weathering a large proportion of PFAS is lost as SFP polymer due to deterioration and loss of textile fibers as well as degradation of the SFP, which ultimately led to a loss in water repellency (SI Figure S16) and color (Figure 2),

The mechanism by which the textile deteriorates is primarily attributable to sunlight-induced photolysis and photooxidation in the presence of radicals in urban air^44^ (e.g., OH• or NO_*x*_)^45,46^ (see S2.3 in the SI for further details to the PA fiber degradation mechanism). However, TF losses and morphological changes in sealed fabrics, as well as in weathered fabrics subjected to washing and mechanical stressors, indicate that heat, abrasion, and washing can also contribute to the formation of low molecular weight PFAS. Considering that the SFP finish is almost entirely present as a thin polymeric layer on the surface of the fibers, it is likely that fibers and particles with high SFP concentrations lost from yarns close to the textile surface have a strong effect on TF reductions (Figure 2 and SI Figure S14). The high TF losses of 4% to 71% detected by CIC (Figure 3) suggest that a high amount of PFAS in the form of polymeric SFPs are lost with the fiber fragments.

Once released into the environment (e.g., into water or soil) these fibers are likely to undergo further degradation processes.^13^ The SFP finish present on the fiber fragments will degrade over time and will eventually contribute to PFAA emissions (Figure 6b). This transformation is a slow process and environmental degradation half-lives for SFPs are likely to be of the order of decades^41−43^ to centuries.^40^

The photooxidation of PFAA precursors such as FTOHs; fluorotelomer acrylate monomers (FTACs) or perfluoroalkyl sulfonamido alcohols, which occur in SFP-based textile finishes as production impurities, were also important sources of PFAAs (see S2.3 in the SI for further details to further details to the PFAA precursor degradation).^35,5,33^ As shown in Figure 5b, 8:2 and 10:2 FTOHs (precursors to long-chain PFAAs) account for 29% of the extractable PFAS in textiles treated with C_8_F_17_–SFPs (i.e., preweathering). The total quantity of PFAA-precursors measured indirectly by the TOP assay was sufficient to explain the concentration of PFAAs analyzed in textile samples after weathering (Figure 5), but due to wash out the total losses of PFAAs could not be quantified. Thus, it remains unclear if side-chain cleavage occurred to the SFPs during weathering and contributed to losses of PFAAs. Despite this uncertainty, SEM clearly showed surface defects on the SFP coating, which resulted in flaking of the fiber surface layers. This flaking suggests that some form of degradation of the SFP backbone occurred during weathering.

The selected conditions of this real time weathering experiment present a worst-case user scenario for functional textiles and it is likely that the observed losses do not occur with a linear decrease over time (see discussion of uncertainties in SI Table S5). These processes might occur gradually under the formation of smaller fiber surface defects first and a stronger decease in TF after continuous weathering when larger fiber breakage and loss takes place. Nevertheless, the emission pathways of textile fragment loss and transformation and release of low molecular weight PFAS residuals revealed in this study, will occur to some extent for textile products with fluorinated treatments and contribute to emissions and accumulation of persistent PFAS in the environment.^14^ Beside the contribution to the use phase related to emissions from weathering, PFAS may also be released during other life cycle phases of functional textiles, for example, during the finishing process in production or at the end of life, for example, during landfilling or waste incineration.^47^ Consequently, the use of fluorinated finishes should be limited to so-called “essential uses”, that is, textile applications that protect health or safety^48^ where currently no alternatives are available.^49,7^

## Supporting Information Available

Additional information including figures, tables, and experimental details and results mentioned in the text (PDF)
