# Supplementary material for: An Outdoor Aging Study to Investigate the Release of Per- And Polyfluoroalkyl Substances (PFAS) from Functional Textiles
Source: Environ Sci Technol. 2022 Feb 25;56(6):3471–9. doi: 10.1021/acs.est.1c06812 (PMC8928479; doi:10.1021/acs.est.1c06812)
Supplement: Supplementary file 1 — es1c06812_si_001.pdf [file es1c06812_si_001.pdf]

An outdoor aging study in Australia to investigate the release of per- and polyfluoroalkyl substances (PFASs) from functional rain jackets

*Steffen Schellenberger,[a,b] Ioannis Liagkouridis,[a,c] Raed Awad[a,c] Stuart Khan,[d] Merle Plassmann,[a] Gregory Peters,[d,e] Jonathan P. Benskin[a] and Ian T. Cousins[a]**

[a]Department of Environmental Science, Stockholm University, SE-106 91 Stockholm, Sweden

[b]RISE Research Institutes of Sweden, Stockholm 111 21, Sweden

[c]IVL Swedish Environmental Institute, 114 28 Stockholm, Sweden

[d]School of Civil and Environmental Engineering, University of New South Wales, Australia

[e]Department of Technology Management and Economics, Chalmers University of Technology, Sweden

Corresponding author, email: Ian.Cousins@aces.su.se

Methods: Detailed descriptions of materials and methods for treatment of fabrics with SFPs (Figure S1; Figure S2 and Table S1); detailed information about the weathering experimental setup including fabric holders (Figure S3) and sampling strategy (Figure S4), meteorological data (Table S2 and Table S3) and a discussion of uncertainty (Table S4) ; additional details to the SEM investigation (Table S6), TF measurements and targeted analysis of PFAAs and PFAA-precursors; list of standards and reagents; information on the statistical analysis, and information on the evaluation of material performance after weathering (Figure S5, Table S5, Table S7 and Table S8).

Results: Microscopic characterization and color fading of fabrics with SFP treatments before and after weathering (Figure S6 to Figure S12), additional data on the TF analysis (Figure S13 to S15 and Table S9-S15), additional data on the targeted PFAS analysis (Table S16-S20), and detailed description on the change of surface density (Figure S17) and degradation mechanism.

Content

Pages: 40 Tables: 21 and Figures: 17

S 1. Experimental 4

S 1.1. Total fluorine determination (detailed method description) 4

S 1.2. Targeted analysis of PFAS (detailed method description) 4

S 1.3. TOP assay (detailed method description) 5

S 1.4. Analysis of fluorotelomer alcohols in textile extracts (detailed method description) 6

S 1.6. Statistical analysis strategy for CIC measurements *6*

S 1.7. Evaluation of material performance after weathering (detailed method description) *7*

Figure S1. SFP-based DWRs with different side-chain modifications 7

Figure S2. Equipment used for fabric`s treatment 8

Figure S3. Schematic of fabric holder modules with different of fabrics. 8

Figure S4. Schematic representation of our sampling strategy to conduct CIC measurements 9

Figure S5. Representation of the ISO 4920 water repellency tests 9

Table S1: Standards and reagents of all monitored PFAS and their internal recovery standards analyzed by LC MS/MS. 10

Table S2. Formulation and application details for the SFP-based DWRs 11

Table S3. Summary of meteorological data 11

Table S4. Comparison of selected weathering data to the conditions used in the Florida weathering test for textiles 12

Table S5. Uncertainty of the weathering experiment and relations to real user scenarios 12

Table S6. Details to abrasion and washing 13

Table S7. Scanning electron microscopy (SEM) parameters 13

Table S8: Explanations to the spray ratings of ISO 4920 water repellency tests 13

Table S9: Explanations to the Grey scale test (ISO 105 A02: 1995) 14

S 1. Results 14

S 2.1 Colour fading post-weathering (detailed result description) 14

Figure S6. SEM overview picture of a PA fabric with C_6F_{13}-SFP treatment after an exposure time of 6 months 15

Figure S7. SEM picture of a PA fabric with C_6F_{13}-SFP treatment after an exposure time of 6 months and additional abrasion and washing. 15

Figure S8. SEM picture of a PA fabric with a C_8F_{17}-SFP treatment after an exposure time of 6 months and additional abrasion and washing. 16

Figure S9. Colour shading of a SEM picture of a PA fabric with C_4F_9-SFP treatment 16

Figure S10. Fabric with C_8F_{17}-SFP after 6 months of weathering and 3000 rubs in the Martindale abrasion resistance test 16

Figure S 11. SEM pictures of the PA rip-stop weave treated with a C_4F_9-SFP finish at different times during the weathering experiment 17

Figure S 12. SEM pictures of the PA rip-stop weave treated with a C_8F_{17}-SFP finish at different times during the weathering experiment 18

Figure S13. Fluorine content of 9 random CIC measurements of a PA fabrics with C_8F_{17}-SFP treatment 19

Figure S14. Calibration curves determined before and after each set of CIC measurements of different timepoints for (a1) the C_4F_9-SFP, (a2) the C_6F_{13}-SFP and (a3) the C_8F_{17}-SFP. (a4) shows measurements of a certified reference material BCR 461 19

Figure S15. PFAA concentrations in untreated fabrics (without SFPs) that were exposed for 6 months in Australia 20

Figure S16. Water repellency before and after weathering 20

Figure S 17. (a) Change of surface density during the weathering experiment for C_6F_{13}-SFPs treated fabrics. 21

S 2.2 Change of surface density (detailed explanation) 22

S 2.3 Degradation mechanism 23

Table S10: Set of CIC measurements for fabrics with C_4F_9-SFPs 25

Table S11: TF concentrations for fabrics with C_4F_9-SFPs 26

(based on calibration curves in Figure S14 a1) 26

Table S12: Set of CIC measurements for fabrics with C_6F_{13}-SFPs 27

Table S13: TF concentrations for fabrics with C_6F_{13}-SFPs 28

Table S14: Set of CIC measurements for fabrics with C_8F_{17}-SFPs 29

Table S15: TF concentrations for fabrics with C_8F_{17}-SFPs 30

Table S16: Statistics for the CIC measurements for fabrics with C_4F_9-SFPs 31

Table S17: Statistics for the CIC measurements for fabrics with C_6F_{13}-SFPs 32

Table S18: Statistics for the CIC measurements for fabrics with C_8F_{17}-SFPs 33

Table S19. Targeted analysis of PFAAs in untreated fabrics. 34

Table S20. Targeted analysis of PFAAs in fabrics with C_4F_9-SFPs. 35

Table S21. Targeted analysis of PFAAs in fabrics with C_6F_{13}-SFPs. 36

Table S22. Targeted analysis of PFAAs in fabrics with C_8F_{17}-SFPs. 37

Table S23. Concentration of FTOHs measured in extracts of unexposed fabrics with C_8F_{17}-SFPs and conversion to fluorine content 38

Table S21: Summary of fluorine content calculations 38

References 39

S 1. Experimental

S 1.1. Total fluorine determination (detailed method description)

Total fluorine analysis was carried out by CIC using an AQF-2100H combustion unit (Mitsubishi, Japan) which was coupled to a Dionex™ ICS-2100 Integrion IC (Thermo Scientific, USA) described in more detail by Schultes et al.[1] In short, fabric pieces were cut and the mass was determined with a balance (model: Sartorius M5P accuracy 0.001 mg). Then fabric samples were placed into ceramic sample boats, which were heated slowly to 1100°C under a flow of oxygen and argon. All combustion gases were trapped in MilliQ water before injection onto an ion exchange column (AS19 Dionex IonPac, Thermo Scientific, USA). Chromatographic separation was achieved using a hydroxide eluent, ramped from 8 to 100 mM. Samples were quantified using a linear calibration curve of NaF (n=5, 1-200 µg/mL 1/x weighting). Calibrations undertaken before and after each set of measurements showed good consistency (Figure S13 a1 to a3 in the SI). The detected amount of fluorine (in µg) in the fabric samples was related to their mass and thus the total fluorine loss caused by weathering could be estimated after different exposure times (see Figure S13. a1 to a4). Measurements of each SFP modification (C_4F_9, C_6F_{13} and C_8F_{17}) and different exposure times were determined in one set of CIC measurements, starting and ending with a calibration curve. This procedure ensured that no change in sensitivity of fluorine detection occurred during the measurement (Figure S13 a4 in the SI). Moreover, a certified reference material, BCR 461 (fluorine in clay with a concentration 568 µg F/g) was measured (n=3 receptions) together with each set of SFPs. Within the different set of measurements an excellent agreement with the certified concentration was detected. Recoveries were 548 µgF/g (96%) for the C_4F_9-SFPs; 553 µgF/g (97%) for the C_6F_{13}-SFPs and 542 µgF/g (95%) for the C_8F_{17}-SFPs (see Figure S13 a4 in the SI for further details).

S 1.2. Targeted analysis of PFAS (detailed method description)

Textile samples were extracted and analyzed as follows. A 1cm^2 piece of textile was cut and put into a 13 mL PP tube. One ng (50 µL of 20 pg/µL) of a mixture of isotopically labelled internal standards (ISTD) was spiked (see Table S1). Extractions took place by addition of 5

mL of methanol, vortexing and sonication for 15 min. The supernatant was transferred to a new 13 mL PP tube and the extraction was repeated. The combined extracts were evaporated to 1 mL under nitrogen and 1 ng (50 µL of 20 pg/µL) recovery standard (RSTD) was added (see Table S1).

Instrumental analysis was carried out using an Acquity UPLC coupled to a Xevo TQ-S triple quadrupole mass spectrometer (Waters) operated in negative ion electrospray ionization, selected reaction monitoring mode.[2] Extracts were chromatographed on a BEH C18 analytical column (2.1×50mm, 1.7 µm particle size, Waters) operated at a flow rate of 0.4mL/min, using a mobile phase composition of 90% water/10% acetonitrile containing 2 mM ammonium acetate (solvent A) and 5% water/95% acetonitrile containing 2 mM ammonium acetate (solvent B). The LC-gradient started with 10% B (held for 0.5 min), ramped to 80% B at 5 min and further to 100% B at 5.1 min (held for 3 min), followed by an equilibration time of 2 min at 10% B. All precursor/product transitions monitored for each analyte can be found in Table S1. Quantitative determination of target compounds was carried out by isotope dilution of an internal standard approach using a linear calibration curve with 1/x weighting. The resulting concentrations were blank subtracted, using the procedural blank that was run in triplicate with each batch of samples (C4, C6 and C8 DWR, respectively). Limit of detections (LODs) were calculated by taking three times the standard deviation of the procedural blank signals. The LODs are listed together with the data in Tables 20, Tables 21 and Tables 22.

S 1.3. TOP assay (detailed method description)

The TOP assay was adapted from the one described by Houtz and Sedlak (2012).[3] Briefly, the extractions of textiles were the same as described above for the PFAS analysis, except for the addition of the ISTD and RSTD. The concentrated extracts of 0.5 mL were filtered using a centrifuge filter and transferred to a 50 mL Falcon tube. This was left opened over night to totally evaporate the methanol. Then, 30 mL Milli-Q water, 0.48 g of potassium persulfate and 0.456 mL of NaOH (10M) were added, the solution was vortexed and placed into an oven at 85 oC for 6 hours. Following this, 0.45 mL of HCl (33%) and 4 ng of ISTD (200 µL of 20 pg/µL) were added. This solution was then extracted using solid phase extraction with Oasis WAX (6 cc, 150 mg, 30 µc). The cartridges were conditioned using 4 mL of 0.1 % NH4OH in MeOH, 4 mL of MeOH and 2 mL of Milli-Q water. The samples were then loaded at ca 1 drop/s under constant vacuum. This was followed by a cleaning step with 4 mL of Milli-Q water. The analytes were eluted from the cartridges with 4 mL of 0.1% NH_4OH in MeOH and 4 mL of MeOH. The combined eluates were concentrated to 0.5 mL under nitrogen and 4 ng of RSTD (20 µL of 200 pg/µL) were added.

Parallel to the textile extracts, additionally three blanks (10 mL of MeOH) and three positive quality control samples (10 mL of MeOH spiked with 5 ng total of EtFOSAA) underwent the same TOP assay and SPE extraction as described above. The recoveries of the positive quality control samples were calculated by summing up all detected PFAS and comparing it to the spiked amount of EtFOSAA. The average recovery was 109.9 % (with a relative standard deviation of 2.1 %), which showed a very good efficiency of the applied TOP assay and SPE extraction. LODs for the TOP assay analysis were calculated either by the lowest calibration standard included in the calibration curve with a signal to noise of at least 3 or by the average blank signal plus three times the standard deviation of the blank signal, whichever was higher.

S 1.4. Analysis of fluorotelomer alcohols in textile extracts (detailed method description)

Textile extracts of unexposed fabrics were also analyzed for 8:2 and 10:2 fluorotelomer alcohols using an ultrahigh performance liquid chromatograph Dionex UltiMate 3000 coupled to a high-resolution mass spectrometer (HRMS) Q Exactive HF Orbitrap (Thermo Scientific). Ten µL of extracts were injected onto a BEH C18 column from Waters (2.1 × 50 mm, 1.7 µm), and eluted using a short gradient starting with 30% water and 70% methanol increased to 100% methanol at 0.5 min (constant over 2 min) and an equilibration at 70% methanol for another 2 min. The HRMS was run with an electrospray ionization in negative mode using a spray voltage of 3.5 eV, sheath gas of 40 arbitrary units (au), aux gas of 10 au, capillary and aux gas temperature at 250 °C. Detection was conducted using a full scan – data dependent MS2 experiment with an inclusion list containing fluorotelomer alcohols. The full scan was run at a resolution of 120 000 (at 200 m/z), an AGC target of 3e6 and a scan range of 100 – 1500 Da. The dd-MS2 was run with a resolution of 15 000 (at 200 m/z), an AGC target of 1e5 and isolation window of 0.4 Da.

A four-point calibration curve containing 8:2 FTOH and its labelled 13C2-8:2 FTOH was run together with solvent blanks and the sample extracts (spiked with 13C2-8:2 FTOH). Calculation was done relative to the labelled standard. 10:2 FTOH was calculated using the response factor of 8:2 FTOH, thus those concentrations are only semi-quantitative.

S 1.6. Statistical analysis strategy for CIC measurements

Data from exposed and control textiles were evaluated for homoscedasticity, after which a two-tailed t-test was used to test for statistically significant differences between group means. A one-tailed t-test (based on equal variances, if this was the case) was used to compare unexposed and exposed samples. A single factor ANOVA was used to compare the mean val-

ues of unexposed samples and sealed controls which were exposed. Targeted PFAA measurements of textile extracts and TOP assay measurements of textile extracts were included without outlier tests under consideration of their standard deviations. More details to the statistical analysis can be found in Table 16, Table 17 and Table 18.

S 1.7. Evaluation of material performance after weathering (detailed method description)

The change in fabric performance was investigated as additional complementary information to the chemical loss. The change in water repellency after weathering (test with 3 replicates of each fabric treatment) was measured using the Spray Test ISO 492013 (see Figure S5 and Table S7 for further information). The color change due to outdoor exposure was investigated visually using the Grey scale Test (ISO 105 A02: 1995; n=2), which is a common assessment tool used in the textile industry to determine color fading due to aging. The color change is judged in comparison to exposed fabrics to the unexposed control (see Table S8 for further explanations). The rating progresses step-wise from Note 5 (no visual change; best rating) to Note 1 (a large visual change; worst rating).

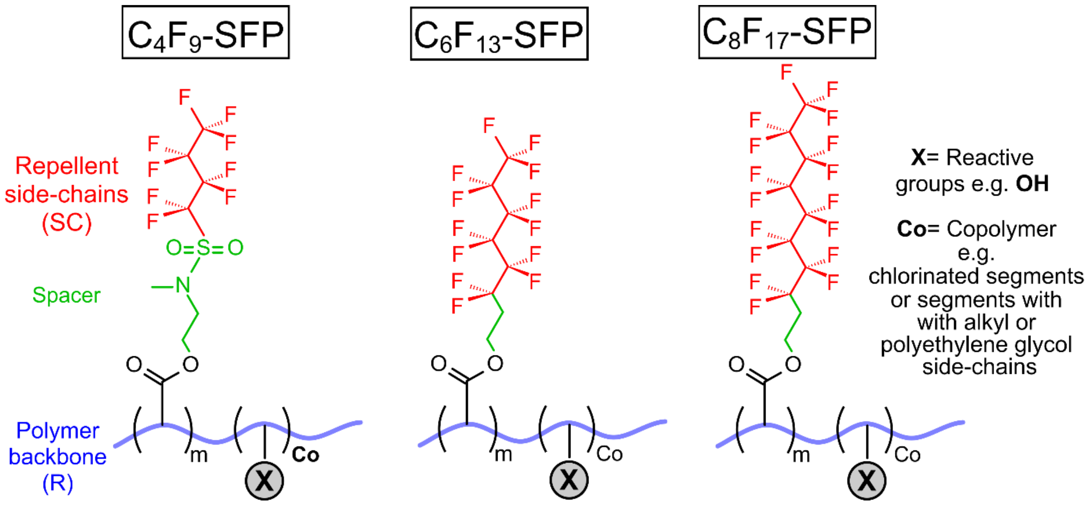

Figure S1. *SFP-based DWRs with different side-chain modifications*

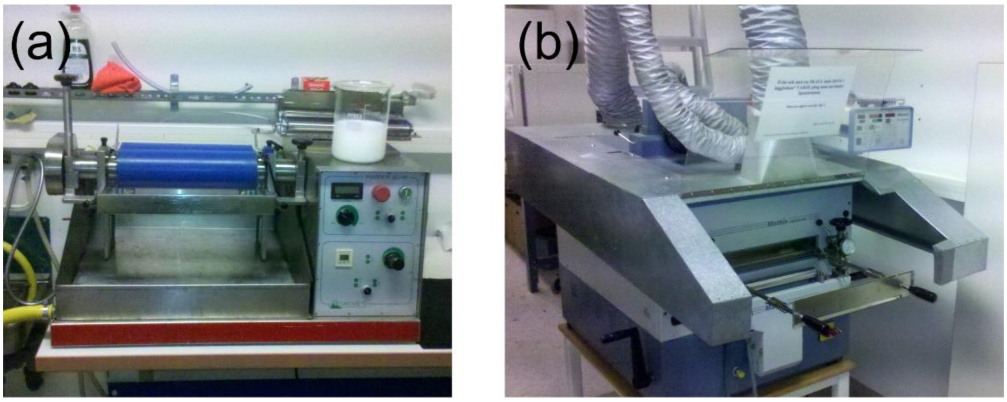

Figure S2. *Equipment used for fabric`s treatment using a pad-dry-cure process with SFPs (a) a foulard for SFP applications and (b) an oven for during and curing*

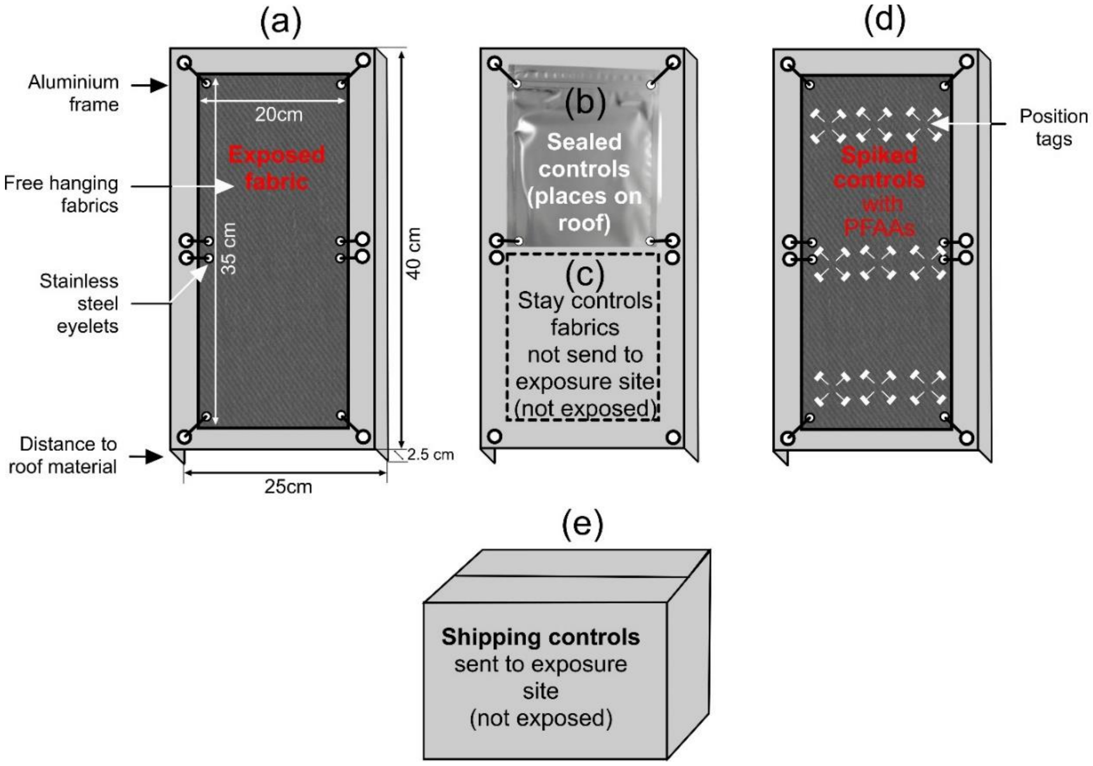

Figure S3. *Schematic of fabric holder modules with different of fabrics. (a) fabrics exposed to weathering including "untreated controls" and (b) "sealed fabric controls" using a zipper bag covered with 3 layers of silver gaffer tap. As (c) "stay controls" a piece of the same fabric stayed unexposed in brown glass flasks excluding UV light and was not shipped to Australia; (d) untreated fabrics that were spiked with PFAAs (spiked controls) and (e) fabric controls that were shipped to Australia but stayed unexposed (shipping controls)*

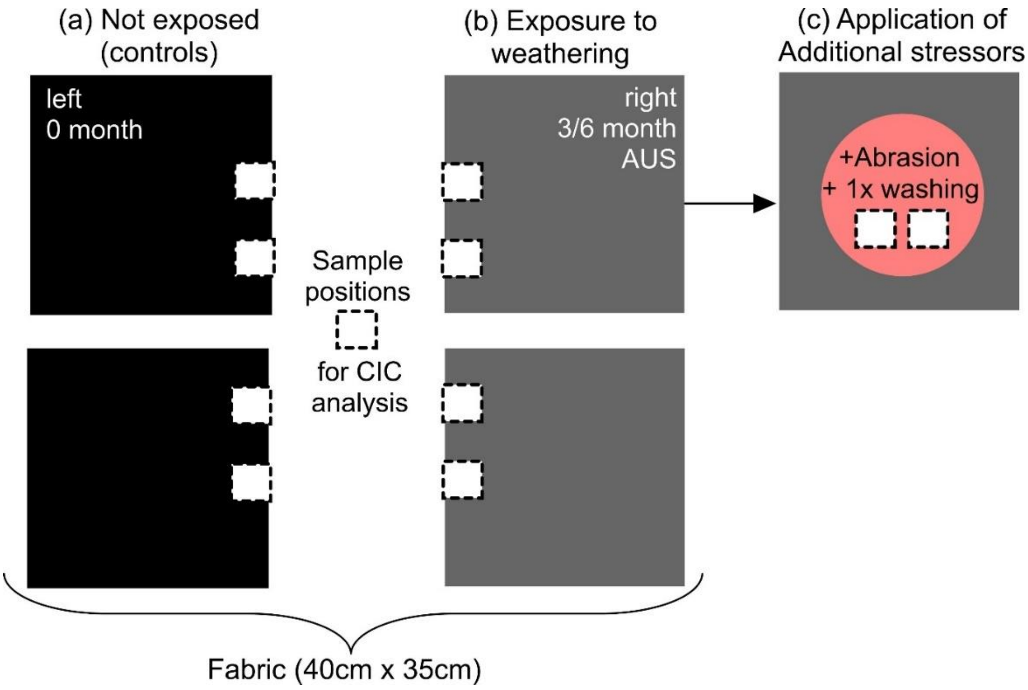

Figure S4. *Schematic representation of our sampling strategy to conduct CIC measurements*

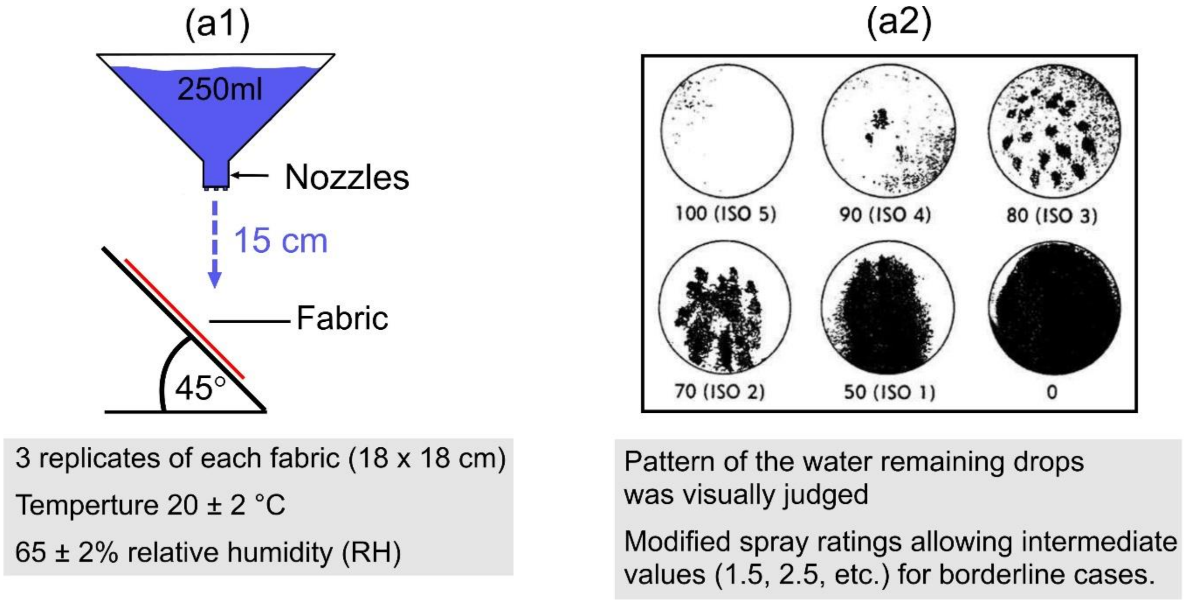

Figure S5. *Representation of the ISO 4920 water repellency tests conducted with SFP treated PA fabrics before and after weathering (a1) represents a scheme of the testing equipment and (a2) are the water repellency ratings according to different wetting patterns (see also Table S8)*

Table S1: *Standards and reagents of all monitored PFAS and their internal recovery standards analyzed by LC MS/MS. All substances were purchased from Wellington Laboratories.*

Target Compounds	Acronym[1]	Quant Ion	Internal Standard	IS Ion
Perfluorobutane sulfonic acid	PFBS	299/80	$^{18}O_2$-PFHxS	403/84
Perfluorohexane sulfonic acid	PFHxS	399/80	$^{18}O_2$-PFHxS	403/84
Perfluorooctane sulfonic acid	PFOS	499/80	$^{13}C_4$-PFOS	503/80
Perfluorodecane sulfonic acid	PFDS	599/80	$^{13}C_4$-PFOS	503/80
Perfluorooctane sulfonamide	FOSA	498/78	$^{13}C_8$-FOSA	506/78
N-Methyl Perfluorooctane sulfonamide	MeFOSA	512/169	d_3-MeFOSA	515/169
N-Ethyl Perfluorooctane sulfonamide	EtFOSA	526/169	d_5-EtFOSA	531/169
Perfluorooctane sulfonamidoacetic acid	FOSAA	556/498	d_3-MeFOSAA	573/419
N-Methyl Perfluorooctane sulfonamidoacetic acid	MeFOSAA	570/419	d_3-MeFOSAA	573/419
N-Ethyl Perfluorooctane sulfonamidoacetic acid	EtFOSAA	584/419	d_3-MeFOSAA	573/419
Perfluoropropanoic acid	PFPrA	163/119	$^{13}C_4$-PFBA	217/172
Perfluorobutanoic acid	PFBA	213/169	$^{13}C_4$-PFBA	217/172
Perfluoropentanoic acid	PFPeA	263/219	$^{13}C_5$-PFPeA	268/223
Perfluorohexanoic acid	PFHxA	313/269	$^{13}C_2$-PFHxA	315/270
Perfluoroheptanoic acid	PFHpA	363/319	$^{13}C_4$-PFHpA	367/322
Perfluorooctanoic acid	PFOA	413/369	$^{13}C_4$-PFOA	417/372
Perfluorononanoic acid	PFNA	463/419	$^{13}C_5$-PFNA	468/423
Perfluorodecanoic acid	PFDA	513/469	$^{13}C_2$-PFDA	515/470
Perfluoroundecanoic acid	PFUnDA	563/519	$^{13}C_2$-PFUnDA	565/520
Perfluorododecanoic acid	PFDoDA	613/569	$^{13}C_2$-PFDoDA	615/570
Perfluorotridecanoic acid	PFTrDA	663/619	$^{13}C_2$-PFDoDA	615/570
Perfluorotetradecanoic acid	PFTeDA	713/669	$^{13}C_2$-PFDoDA	615/570
Perfluoropentaadecanoic acid	PFPeDA	763/719	$^{13}C_2$-PFDoDA	615/570
Perfluorohexadecanoic acid	PFHxDA	813/769	$^{13}C_2$-PFDoDA	615/570
Perfluoroheptadecanoic acid	PFHpDA	863/819	$^{13}C_2$-PFDoDA	615/570
Perfluorooctadecanoic acid	PFOcDA	913/869	$^{13}C_2$-PFDoDA	615/570
4:2 polyfluoroalkylphosphate diester	4:2/4:2 diPAP	589/343	$^{13}C_4$-6:2/6:2 diPAP	793/445
6:2 polyfluoroalkylphosphate diester	6:2/6:2 diPAP	789/443	$^{13}C_4$-6:2/6:2 diPAP	793/445
8:2 polyfluoroalkylphosphate diester	8:2/8:2 diPAP	989/543	$^{13}C_4$-8:2/8:2 diPAP	993/545
10:2 polyfluoroalkylphosphate diester	10:2/10:2 di-PAP	1189/643	$^{13}C_4$-8:2/8:2 diPAP	993/545
3-perfluoropropyl propanoic acid	FPrPA	241/177	$^{13}C_4$-PFOA	417/372
3-perfluoropentyl propanoic acid	FPePA	341/237	$^{13}C_4$-PFOA	417/372
3-perfluoroheptyl propanoic acid	FHpPA	441/337	$^{13}C_4$-PFOA	417/372
4:2 Fluorotelomer sulfonate	4:2 FTS	327/307	$^{13}C_2$ -6:2 FTS	429/409
6:2 Fluorotelomer sulfonate	6:2 FTS	427/407	$^{13}C_2$ -6:2 FTS	429/409
8:2 Fluorotelomer sulfonate	8:2 FTS	527/507	$^{13}C_2$ -6:2 FTS	429/409
8:2 Fluorotelomer unsaturated carboxylic acid	8:2 FTUCA	457/393	$^{13}C_2$-PFDA	515/470
Dodecafluoro-3h-4,8-dioxanonanoate	ADONA	377/85	$^{13}C_4$-PFOA	417/372
9-chlorohexadecafluoro-3-oxanonane-1-sulfonate	9Cl-PF3ONS	531/351	$^{13}C_4$-PFOA	417/372
11-chloroeicosafluoro-3-oxaundecane-1-sulfonate	11Cl-PF3OUdS	631/451	$^{13}C_2$-PFDA	515/470
**Recovery Standards**				
$^{13}C_8$ labeled Perfluorooctanoic acid	M8-PFOA	421/376		
$^{13}C_8$ labeled Perfluorooctane sulfonic acid	M8-PFOS	507/80		

Formic acid, ammonium sulfate, and ammonium acetate were obtained from Merck. Potassium hydroxide was purchased from VWR, and acetonitrile was obtained from Honeywell.

Table S2. *Formulation and application details for the SFP-based DWRs*

No.	Description	Formulation	DWR application conditions
I	C_4F_9-SFP based DWR (short chain technology)	60 g dm^{-3} S-SFP; 30 g dm^{-3} HC-based extender	Drying: 120 °C; Curing: 300 s 160 °C
II	C_6F_{13}-SFP based DWR (short chain technology)	80 g dm^{-3} S-SFP; 10 g dm^{-3} cross linker	Drying: 120 °C; Curing: 35 s 175 °C
III	C_8F_{17}-SFP based DWR (long chain technology)	80 g dm^{-3} L-SFP	Drying: 120 °C; Curing: 35 s 175 °C
All	**Additional information to the SFPs**<br>SFPs and other chemical ingredients in the formulations such as cross linkers and surfactants were also provided by the chemical raw material producers.<br>At the time of this study, the C_6F_{13}-SFP finish was used in products on the market, the specific C_4F_9-SFP finish used was a new product under development and not on the market at the time (other “C4” products were on the market), and the C_8F_{17}-SFP finish was no longer on the market due to the phase-out of long-chain SFPs by manufacturers and regulation within the European Union. It is important to note that “C4”, “C6”, and “C8” are the principal chain lengths of SFP-based industrial textile finishes, but other chain lengths are likely to exist in lower abundances within these emulsions.		
All	**Pad-dry-cure process:**<br>SFPs were applied according to information provided by the chemical suppliers similar to industrial production processes, but working in batch treatment of fabrics. Water-based emulsions of DWRs were applied in a laboratory non-continuous dip coating process (padding), as follows. A piece of fabric (0.14 m^2) was immersed in a 2 L glass beaker, containing 1 L of DWR formulation and kept there for 30 s while stirring with a PTFE-stirring magnet. The excess liquid was removed by using a not continuous foulard equipment (model BVHP, Roaches England; see Figure S 2a) using two runs at 2.9 rpm and a nip pressure of 3.7 bar. After adsorption of DWR polymers to the fibres, the fabrics were dried followed by a curing step in an oven (Discontinuous Labdryer; type LTE; Mathis, Switzerland; see figure S 2b) to promote the crosslinking reaction of the DWR-polymer (additional information sample preparations can be found in Table S2). All SFPs had a good durability after washing, abrasion and artificial weathering as determined in a previous study[1] consistent with commercially manufactured textiles		

Table S3*. Summary of meteorological data during the study period. Insulation and rainfall data were collected 800m from the exposure site while average daily temperatures were collected 6.8 km from the exposure site.*

Summary daily tracked weathering data near the exposure site in Sydney (Australia)											
Exposure period	Start date	End date	Duration [days]	Insolation [MJ/m2]	Insolation per month [MJ/m2]	Rainfall sum [mm]	Rainfall per month [mm]	Max T [°C]	Average max T [°C]	min T [°C]	average min T [°C]
"spring"	2017-08-27	2017-11-26	91	1809	603.0	151.4	50.5	37.3	23.9	6.5	14.2
"summer"	2017-11-27	2018-02-25	91	2059	686.2	127.8	42.6	43.7	28.8	14.9	19.9

Table S4. *Comparison of selected weathering data to the conditions used in the Florida weathering test for textiles*

Comparison to Florida Direct Weathering test (e.g. AATCC TM111 for textiles)							
Australia Average insolation per month [MJ/m2]	644.6	Australia Rainfall per month [mm]	46.5	Australia Florida test Average max T in summer [°C]	28.8	Australia average min T in summer [°C]	19.9
Florida test average insolation per month [MJ/m2]	548.8	Florida test rainfall per month [mm]	140.4	Florida test Average max T in summer [°C]	34.0	Florida test average min T in summer [°C]	23.0

Table S5. *Uncertainty of the weathering experiment and relations to real user scenarios*

**Process**	**Relations to real user scenarios**
Sun radiation	The amount of sun radiation and exposure time on the roof is very high and simulates a worst-case scenario.<br><br>The average solar radiation in Australia was 645 MJ/month higher than e.g. in Sweden (495 MJ/month in average during a comparable time period)
Temperature	Additional heat reflection from surface could be excluded to the greatest extent since the fabric modules cast a shadow on the roof material (coated corrugated iron).<br><br>Nevertheless, the influence of the heating of the roof material could still play a role and temperatures might exceeded the temperatures tracked on the weathering station. It is also unrealistic that repellent garments such as jackets are worn at high temperatures. Due to the type of roof materials elevated roof temperatures might be expected due to the heating of the roof and development of a microclimate close to the roof surface.[4,5]
Exposure time	Was of a normal lifetime
Abrasion and washing	Usually the stress events of weathering, abrasion and washing would occur alternating. Abrasion and washing were applied after weathering. Thus, this does not correspond to a realistic user scenario
Summary of implications	Outdoor exposure testing is a common practice to evaluate the durability of textiles (e.g. color loss, cracking or loss of mechanical properties).[6] Such experiments are less controlled than laboratory weathering experiments, but represent natural, albeit relatively extreme, exposure conditions<br><br>The conditions chosen in the weathering experiment presented here were extreme, especially the high sunlight exposure. The continued exposure of textiles on the rooftop is also not a very realistic user scenario. Functional rain gear is unlikely to be used in sunny weather to the same extent. However, some textiles with SFP finishes such as ski jackets or impregnation on strollers or tents are used more intensively during sunny weather, which makes our experiment relevant. Losses of 50% and more of TF are extreme and are unlikely to occur under normal use of outdoor textiles. It is further likely that the observed TF losses do not occur with a linear decrease over time.

Table S6. *Details to abrasion and washing*

Method	Instrument	Description
Martindale abrasion resistance<br>ISO 12947-2	NU Martindale; James Heal; UK	3000 rubs ~lifetime abrasion<br>Abrasion resistance of the DWR-treated fabrics by rubbing the fabrics in a Lissajous pattern against standard wool (James Heal)
Domestic washing<br>ISO 26330 ISO, 2001	Electrolux; Wascator FDM 71 MP-Lab	1 cycle after abrasion test<br>Temperature: 40 °C;<br>Detergent ECE A<br>PES ballast

Table S7. *Scanning electron microscopy (SEM) parameters to characterize surface defects of the PA fabric before and after weathering*

Method	Instrument	Settings and description
Weave overview and fibre analysis	SEM: Jeol JSM-7000 F FEG-SEM	Voltage 0.8kV<br>Overview weave<br>40x; 100x; 200x
		Fibre analysis (weft and warp yarns)<br>200x; 500x; 1500x

Table S8: *Explanations to the spray ratings of ISO 4920 water repellency tests*

ISO 5	ISO 4	ISO 3	ISO 2	ISO 1
No sticking or wetting of the upper surface	Slight random sticking or wetting of the upper surface	Wetting of upper surface at spray points	Partial wetting of whole upper surface	Complete wetting of whole upper surface

***Table S9:** Explanations to the assessment of colour change of the fabrics using the Grey scale test (ISO 105 A02: 1995)*

Grey scale	Settings and description
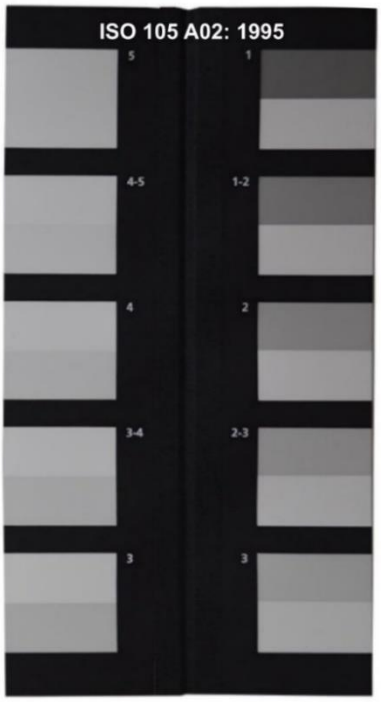	Colour fading of back PA fabrics after outdoor exposure was judged visually in a special colour judgment cabin using day light (D).<br><br>A tool, known as a Grey scale, is used to visually assess and compare the loss of colour of a specimen (see left side)<br><br>Exposed samples were compared to a control (unexposed fabrics)<br><br>Rating goes step-wise from:<br>Note 5 = no visual change (best rating)<br>to<br>Note 1 = a large visual change (worst rating).<br>The Grey scale has the 9 possible values:<br>5, 4-5, 4, 3-4, 3, 2-3, 2, 1-2, 1<br><br>Each pairing in the Grey scale illustrates the difference in shade between a sample which after weathering to a control sample and corresponds to a numbered rating.

S 1. Results

S 2.1 Colour fading post-weathering (detailed result description)

During the weathering experiment the initially black PA fabrics showed color fading and became grey (see Figure S9). The Grey scale test (Table S8) showed a rating of 4, which indicated a small but visible change compared to the control. After 6 months, fabrics showed strong fading and a light grey color (Grey scale rating 1). After abrasion and washing the fabrics became darker again (Grey scale rating 3). The results of the Grey scale testing are also in agreement with increasing fiber surface defects observed by SEM (figure S11 and figure S12). It is likely that the higher surface roughness caused by fiber surface defects caused a more diffuse light scattering at the fiber surface that caused a brighter appearance of the fabrics. Washing and abrasion most likely caused a removal of parts of the surface fibers that underwent weathering and brought up darker fibers from beneath, resulting in a higher gray scale rating (see Figure S10).

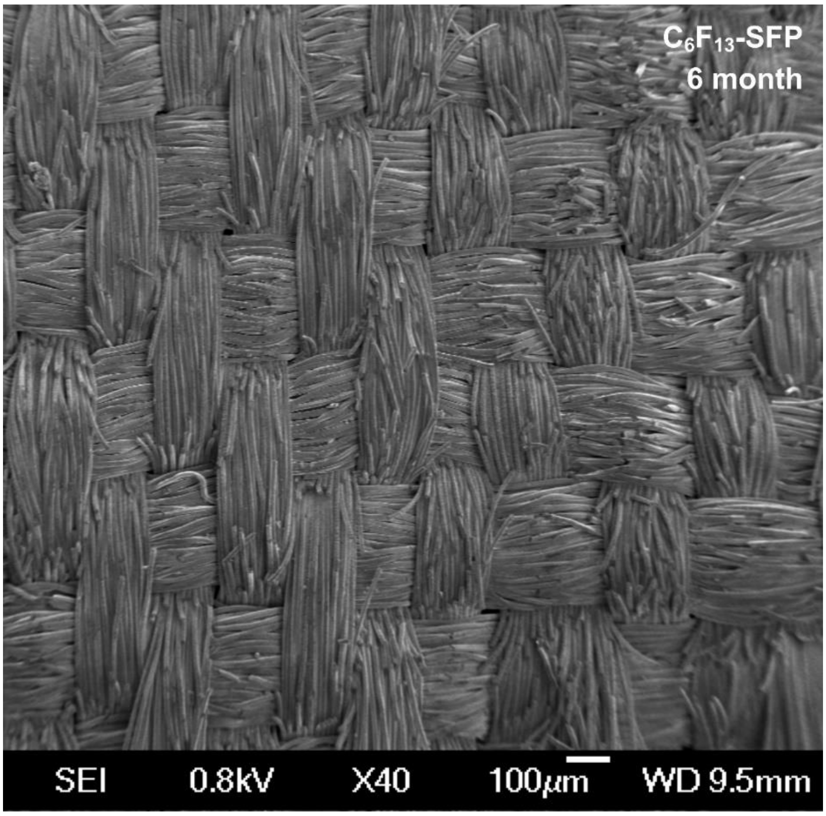

Figure S6. *SEM overview picture of a PA fabric with C_6F_{13}-SFP treatment after an exposure time of 6 months shows that fiber fragments were lost over the entire fabric.*

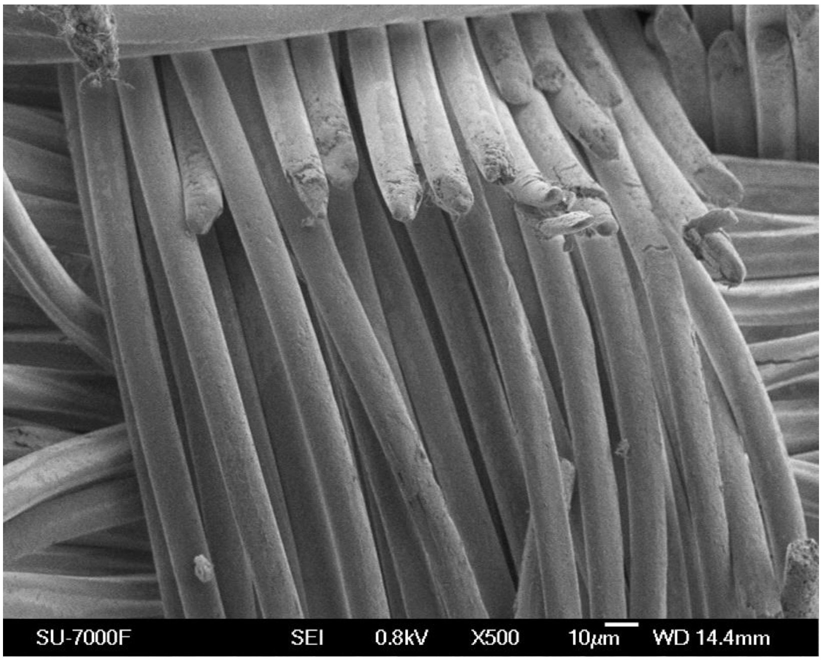

Figure S7. *SEM picture of a PA fabric with C_6F_{13}-SFP treatment after an exposure time of 6 months and additional abrasion and washing. Fibers had rounded ends which proves not only the loss of fiber fragments but also abrasion and loss of smaller particles*

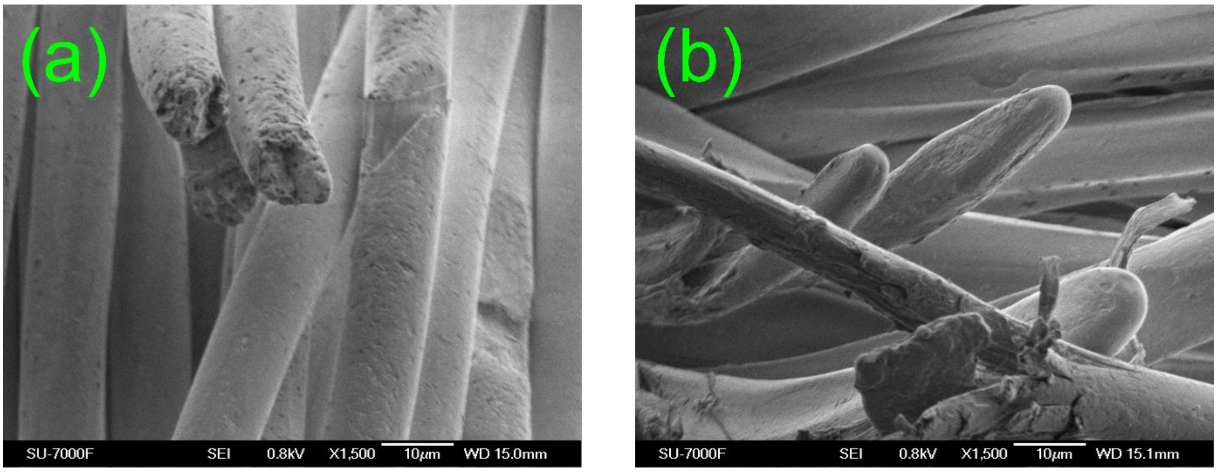

Figure S8. *SEM picture of a PA fabric with a C_8F_{17}-SFP treatment after an exposure time of 6 months and additional abrasion and washing. (a) shows rounded fibers and material defects across the whole core of the fibers (b) shows fibers breakage with delamination and formation of fiber fragments of different shapes.*

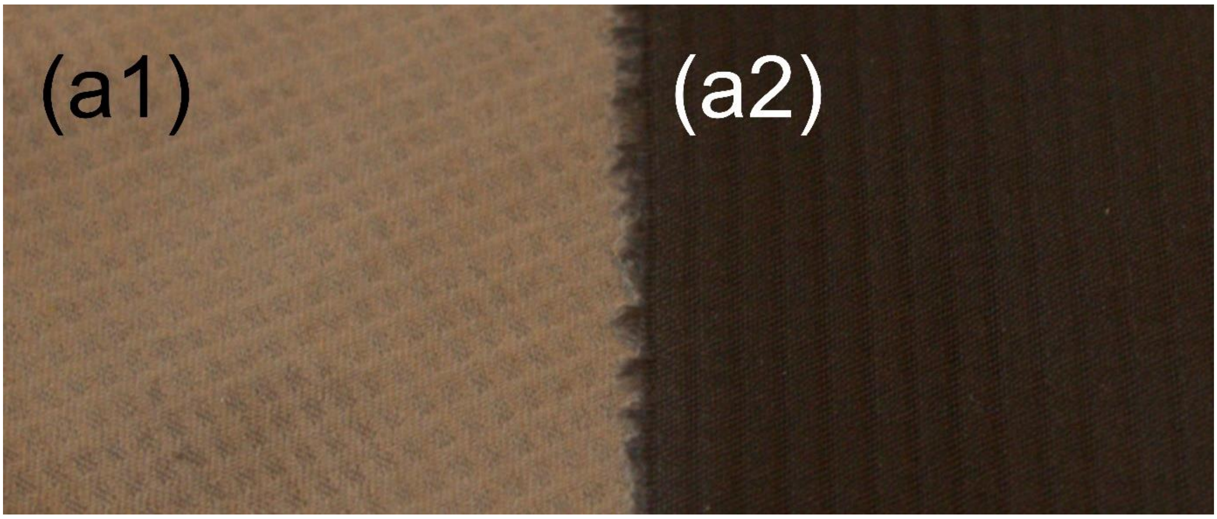

Figure S9. *Colour shading of a SEM picture of a PA fabric with C_4F_9-SFP treatment (a1) exposed side after 6 months and (a2) backside of the same fabric*

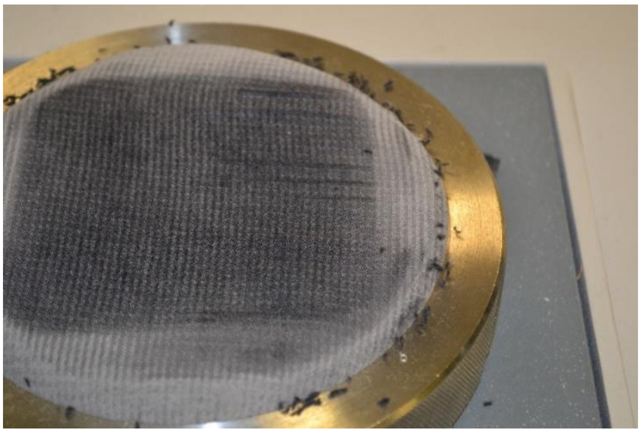

Figure S10. *Fabric with C_8F_{17}-SFP after 6 months of weathering and 3000 rubs in the Martindale abrasion resistance test (ISO 12947-2)*

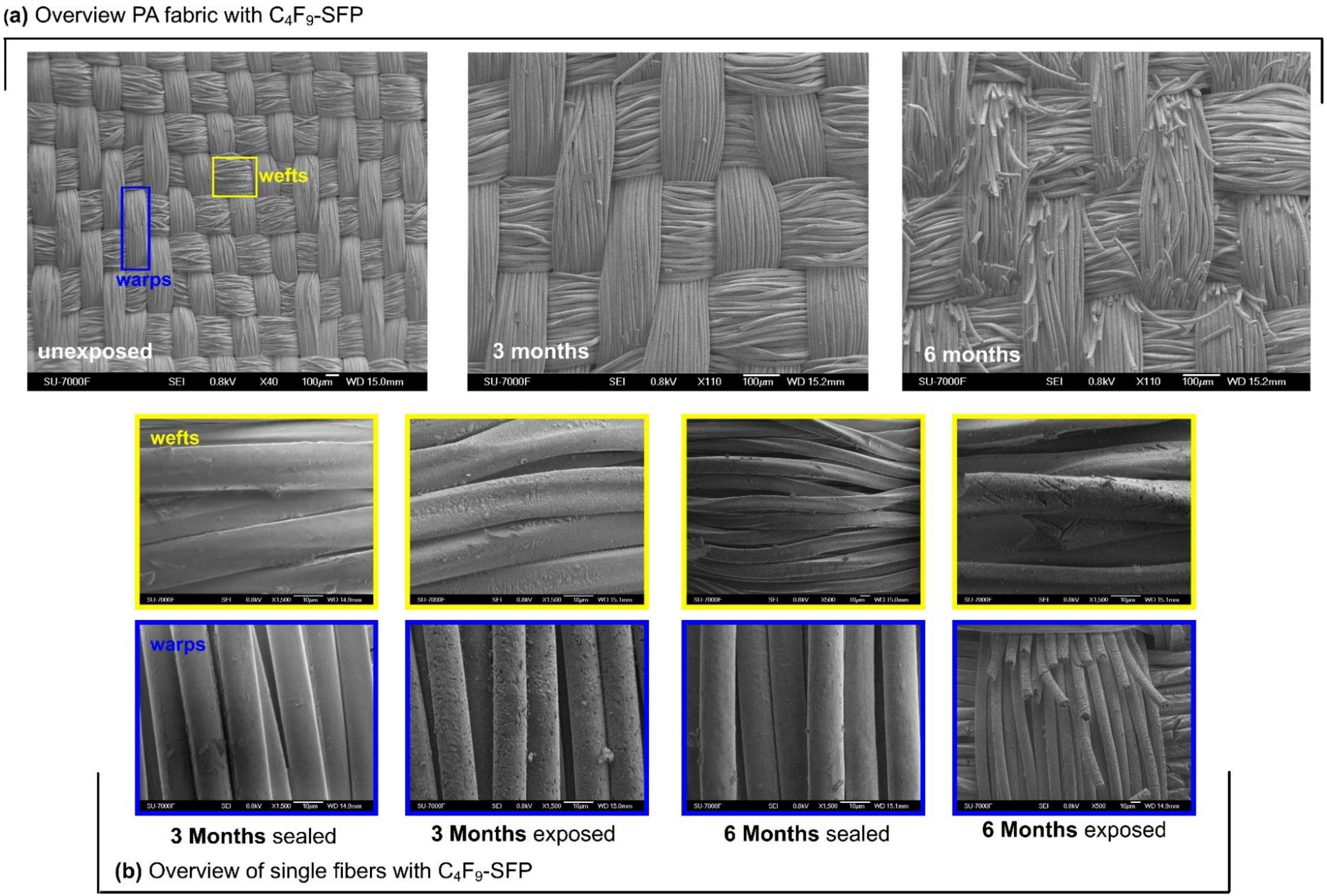

***Figure S 11**. SEM pictures of the PA rip-stop weave treated with a C_4F_9-SFP finish at different times during the weathering experiment*

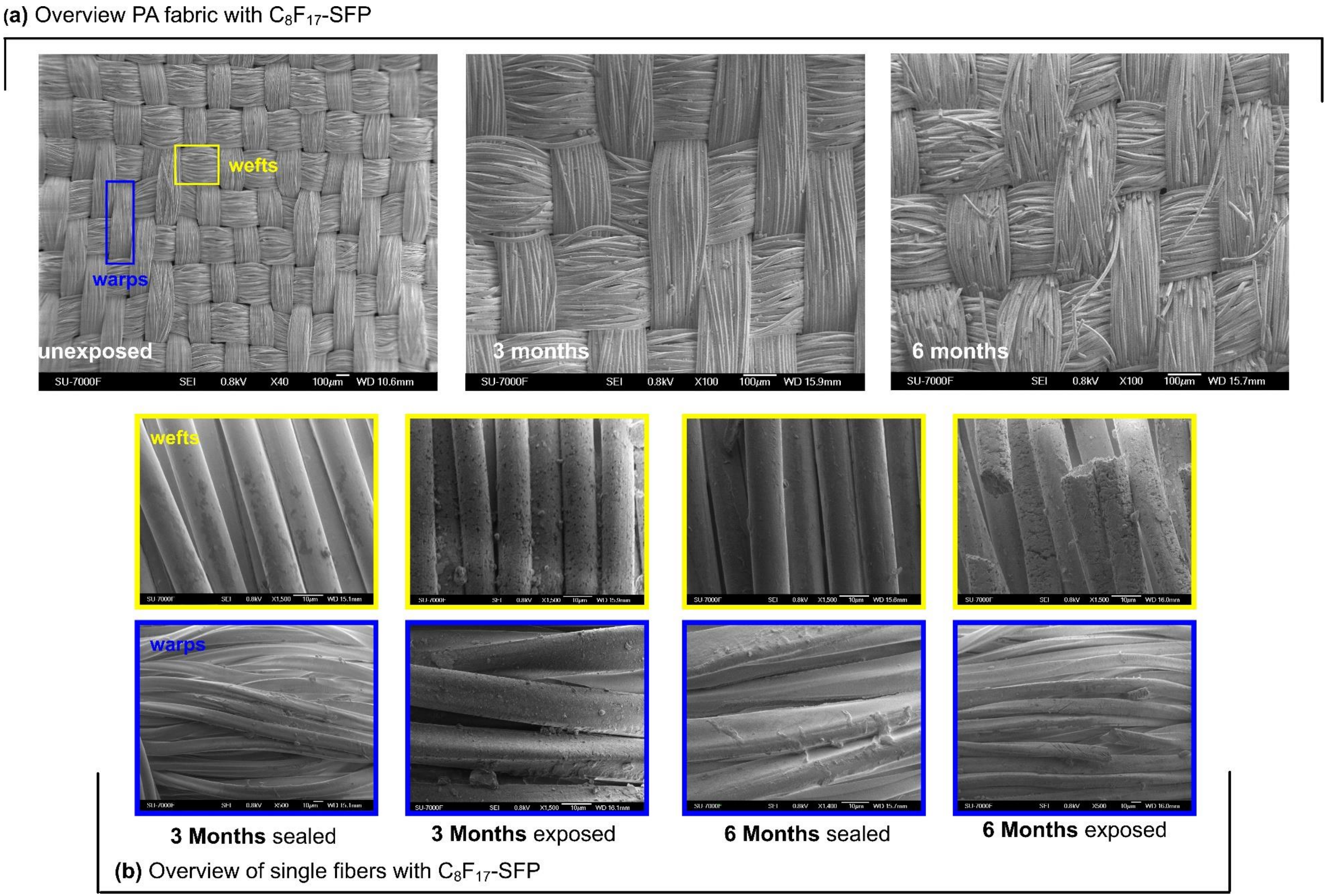

Figure S 12*. SEM pictures of the PA rip-stop weave treated with a C_8F_{17}-SFP finish at different times during the weathering experiment*

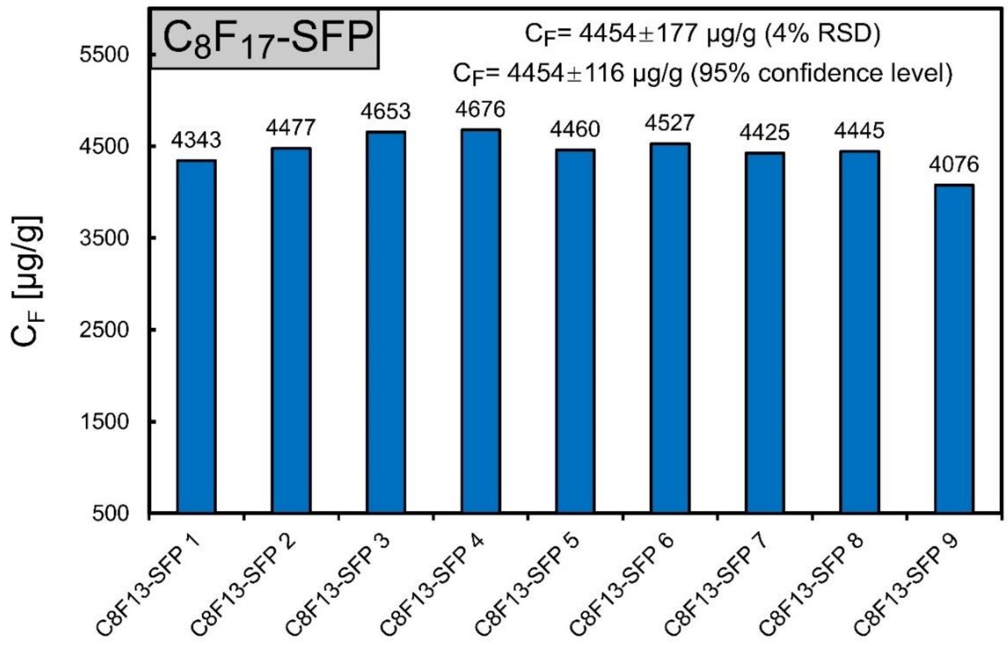

Figure S13. *Fluorine content of 9 random CIC measurements of a PA fabrics with C_8F_{17}-SFP treatment*

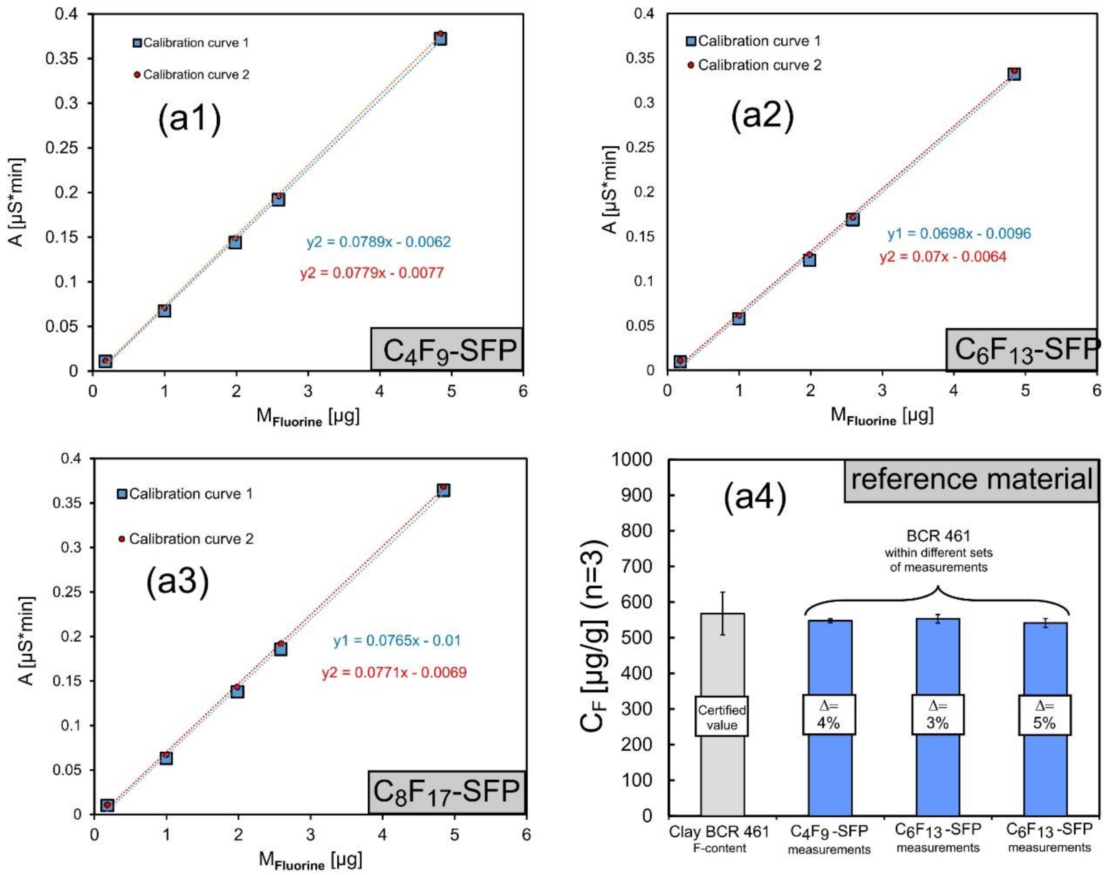

Figure S14. *Calibration curves determined before and after each set of CIC measurements of different timepoints for (a1) the C_4F_9-SFP, (a2) the C_6F_{13}-SFP and (a3) the C_8F_{17}-SFP. (a4) shows measurements of a certified reference material BCR 461 added for each set of measurements for the different textile modification.*

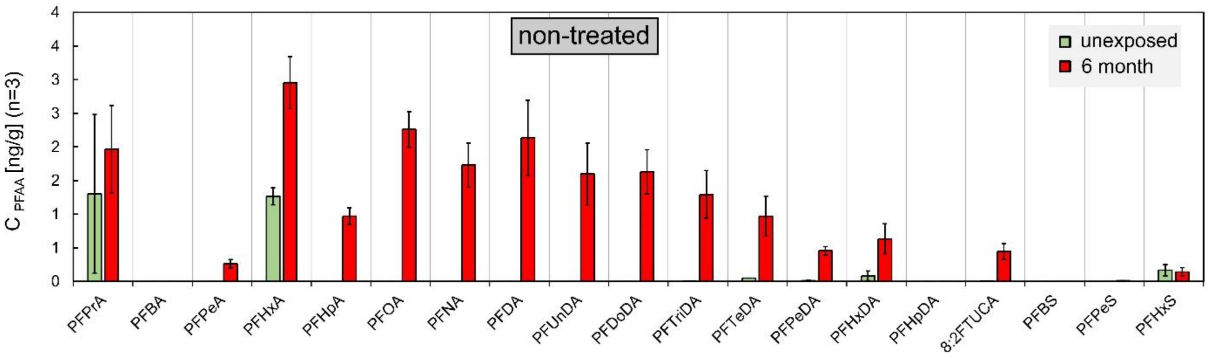

Figure S15. *PFAA concentrations in untreated fabrics (without SFPs) that were exposed for 6 months in Australia*

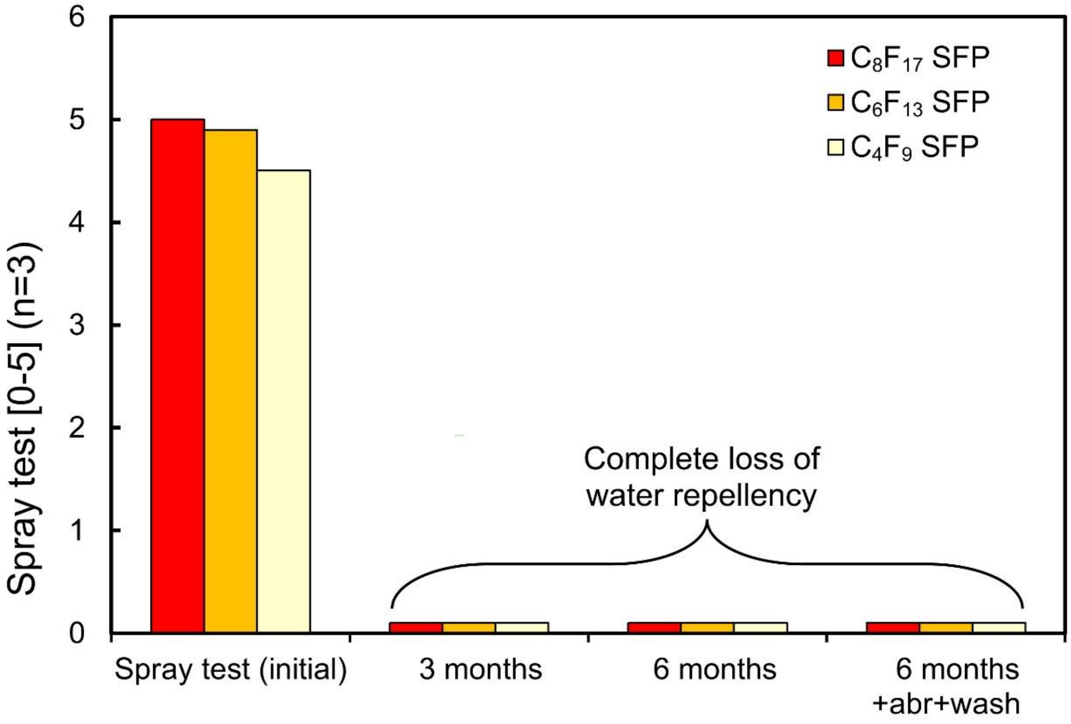

Figure S16*. Water repellency before and after weathering*

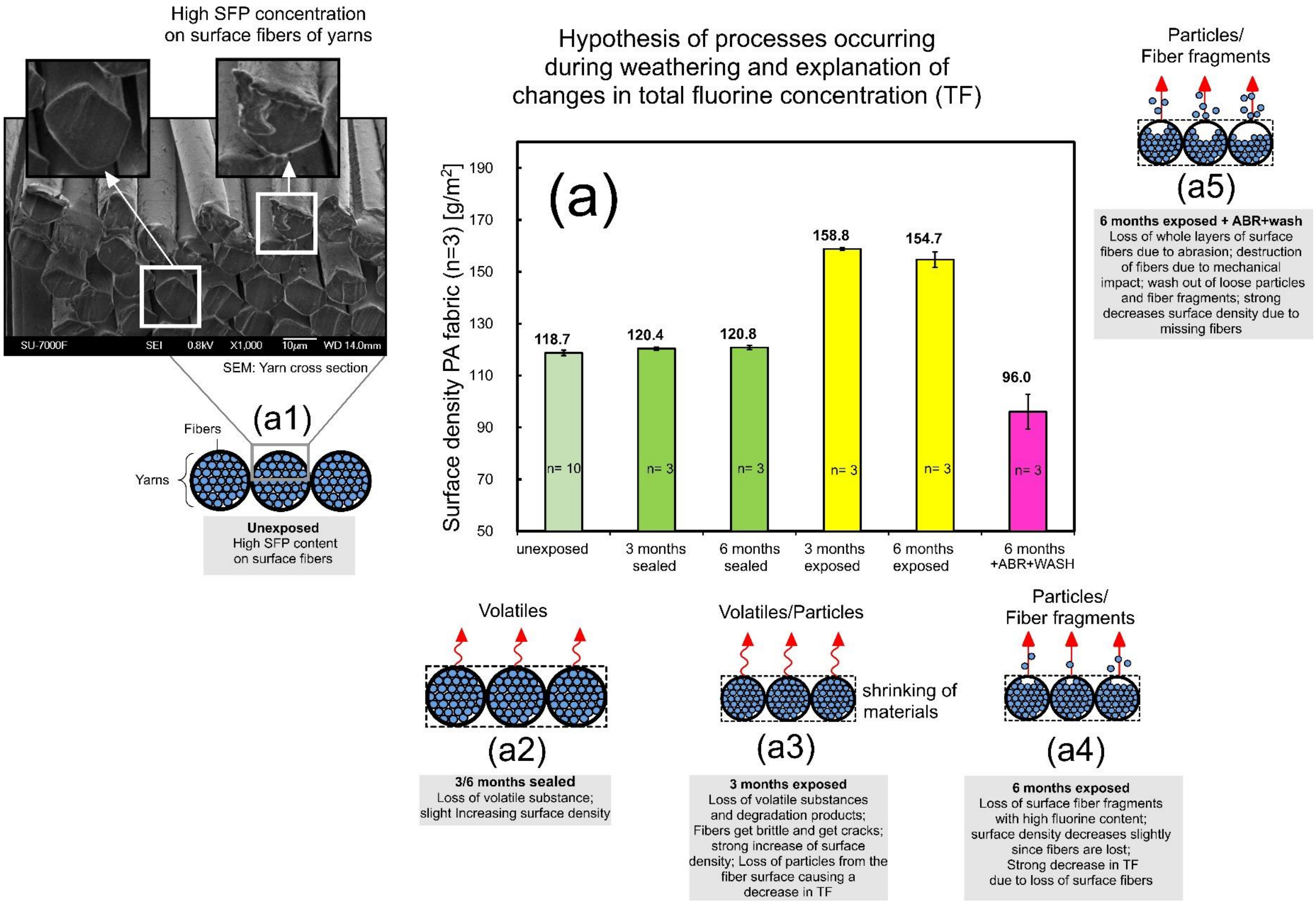

Figure S 17. *(a) Change of surface density during the weathering experiment for C_6F_{13}-SFPs treated fabrics. Processes (a1 to a5) are hypothetical processes that occur and their relation to changes in total fluorine concentration; the SEM picture of a single yarn cross section was taken in a study by the authors[7] using the same C_6F_{13}-SFP treatment and textile finishing conditions.*

S 2.2 Change of surface density (detailed explanation)

An additional effect that was observed during the weathering experiment was the change of surface density of the textile weaves during weathering. Before and after each exposure step fabric pieces were cut-out and the weight per surface area of the fabrics (in g/cm^2) was measured in addition to changes in TF concentrations. As can be seen in the graph in Figure S17 fabrics with C_6F_{13} SFP treatments underwent strong changes in surface density and it is likely that the PA fabrics with the other textile modifications (C_4F_9- and C_8F_{17}-SFPs) underwent comparable changes since fabrics in this study used the same PA base material. As can be seen in Figure S17, fabric surface density slightly increased from ~119 g/m^2 for the unexposed samples to 120 g/m^2 for the exposed textile fabrics that were sealed from sunlight (Figure S17 a2). A strong increase in surface density was measured after 3 months (~159 g/m^2) and after 6 months (~155 g/m^2) of weathering (Figure S17 a3 and a4).

A possible explanation for these changes is a shrinking of the fabrics, which is a common effect observed during the weathering of textiles.[8] It is likely that textile shrinking made yarns (and fibers) move closer together resulting in an increase in surface density (mass per area). Studies have shown that the density of synthetic polymers only undergoes slight changes due to weathering.[9] PA (Nylon 6,6) used in this study might undergo a slight increase in density due to secondary crystallization processes that can occur for these semi-crystalline polymers.[10] This small changes in density, however, cannot explain the high surface density changes observed in this present study.

After applying additional washing and abrasion following outdoor exposure the surface density decreased strongly to ~96 g/m^2 which can be explained with a loss of entire fiber segments (figure S17 a5). Although it is a common procedure to analyze chemical losses for textiles based on surface area, it is more meaningful to discuss TF losses on a weight basis as done in this study.

S 2.3 Degradation mechanism

Further details to the PA fiber degradation:

Further details to the PA fibre degradation: It is likely that sunlight-induced photooxidation in the presence of radicals in urban air[11] (e.g. OH• or NO_x) have been the main cause of molecular degradation in the form of polymer chain scission of the PA polymers (Polyamide 6,6). This can lead to a brittle top layer of fibers that can break easily.[12] In addition, semi-crystalline polymers such as PA can undergo a secondary crystallisation near the surface due to weathering resulting in a surface layers with a higher density than the material below.[13] This causes stress within the material and enhances the formation of cracks. The shrinking of the material and change in surface density (Figure S17) is an indication that PA materials underwent strong changes in material properties during weathering. With continuous photo-aging and mechanical stress through e.g. movement of the free hanging fabrics (see fabric holder Figure S3.) cracks are formed on the fiber surface that grow deeper into the material causing facture and eventually fiber breakage.[13]

Fabric abrasion using the Martindale method (Table S6) resulted in complete destruction of fabric materials and large parts of surface fibers and removal of yarns (Figure S10). Additional washing may have also washed a fraction of loose particles and fiber fragments from the inner areas of the weave. This large impact on the materials could be supported by SEM microscopy (e.g. Figure S7 and Figure S8) and gray scale measurements (Figure 2 in the main text). The very high TF losses after abrasion and washing are likely to be caused by removal of upper fiber layers, but must be also caused by a wash off of SFP particles from the inner textile structure to explain the high TF reductions. A study by Schellenberger et. al.[7] has shown that related losses of micro-fibers with fluorinated SFP treatments can occur during washing of functional textiles.

These exposure conditions represent extreme use scenarios for a functional rain jacket and the high TF losses therefore represent a worst-case scenario. It is likely that the high reduction of TF concentrations after weathering is caused by the loss of textile fibers and particles with high SFP content from the textile surfaces. The SEM-pictures suggest that especially after 6 months a large quantity of fiber fragments are lost from surface yarns (Figure S11 and Figure S12) and after washing and abrasions entire layers of fibers were lost. Previous investigations using related DWRs and fabric materials have shown that SFP finishes were almost entirely present on top of upper textiles fibers (Figure 17a1). The removal of entire layers of surface fibers caused by abrasion and wash out of loose particles during washing might explain the even higher losses in TF for these samples.

Further details to the PFAA precursor degradation:

A number of processes are known to contribute to the occurrence of low molecular weight PFAS from SFP-coated textiles. PFAAs and their low molecular weight precursors (e.g. fluorotelomer alcohols (FTOHs)) are known to occur as residual impurities on SFP-coated textiles.

Smog chamber studies simulating this process have shown that OH-radicals mediated atmospheric oxidation.[14] PFAS with the formula $CF_3(CF_2)_nCH_2CH_2X$ (where X is the functional group) were shown to first form the corresponding fluorotelomer aldehydes (FTALS) as main degradation products which further degraded to form perfluorocarboxylic acids (PFCAs). During the oxidation of 8:2 FTOH a homologous series of shorter chain PFCAs was detected. This observation leads to the hypothesis that the formation of different PFAAs in our weathering study could also be caused by related processes. Thus, the oxidation of different low molecular weight PFAS in the presence of radicals such as OH• or NO_x• in urban air might be an explanation for the elevated levels of PFAAs. The spiking experiment suggested that a fraction of the PFAAs formed during weathering are washed-out from the textiles during rain events and that actual amount of PFAAs formed during weathering is higher than the measured amounts. Results are not directly transferable, however, since PFAAs were spiked on untreated fabrics. Water soluble PFAS formed in textiles treated with fluorinated DWR finishes might have a higher affinity to the SFPs[15] present on the textile surface and may be washed off at a lower rate.

Wash-off might also occur for water soluble intermediates (e.g. fluorotelomer carboxylic acids) Also, volatile PFAA precursor such as FTOHs or perfluoroalkyl sulfonamides might be formed during weathering and lost into air especially due to the high temperatures (Tmax were >40°C) on the roof. Van der Veen et al.[16] found increased levels of different FTOHs after a weathering study with related textiles indicating that the formation of volatile intermediates is a relevant process.

Domestic washing may contribute to emission of these residuals into waste streams as well as the release of microplastics (i.e. textile fibers) containing SFPs.[7] In addition to contributing to environmental microplastic pollution, fibers with SFP[17] coatings may be cleaved into smaller units,[18,19] e.g. through exposure to UV radiation and get a source of PFAAs caused by long term degradation processes in the environment.

***Table S10:** Set of CIC measurements for fabrics with C_4F_9-SFPs*

Sample	**Area [µS*min]**	
boat blank 1	0,0006	before calibration
boat blank 2	0,0005	
boat blank 3	0,0007	
boat blank 4	0,0021	during measurements
boat blank 5	0,0019	
boat blank 6	0,0013	
boat blank 7	0,0012	
boat blank 8	0,0014	
boat blank 9	0,0009	
boat blank 10	0,0013	
boat blank 11	0,0026	after calibation 2

Note: 100µl=0.1g was dosed to the boats

**Calibration curve 1**		**Area [µS*min]**	$C_{corrected}$ [ppm]	Mass Deviation [%]	$Mass_F$ [µg]
	2 ppm NaF	0.011	1.79	90.05	0.18
	10 ppm NaF	0.0673	9.97	100.48	1.00
	20 ppm NaF	0.1441	19.84	198.42	1.98
	25 ppm NaF	0.1919	25.90	103.58	2.59
	50 ppm NaF	0.3722	48.45	96.90	4.84

**Fabric mesurments**		**Area [µS*min]**	Weigt fabrics [mg]
S235	stay control-AM (PA1)	0.3332	0.859
S236	stay control-AM (PA 1)	0.3359	0.86
S237	stay control-AL (PA 1)	0.2327	0.652
S238	stay control-AL (PA 1)	0.1802	0.598
S239	shipping control-AM (PA 1)	0.3284	0.852
S240	shipping control-AM (PA1)	0.3654	0.978
S241	shipping control-AL (PA 1)	0.2388	0.596
S242	shipping control-AL (PA 1)	0.3445	0.831
S243	3 monthss sealed-AM (PA 2)	0.1611	0.477
S244	3 monthss sealedAM (PA 2)	0.1404	0.408
S245	3 monthss sealed-AL (PA 2)	0.1158	0.387
S246	3 monthss sealed-AL (PA 2)	0.1334	0.904
S247	6 monthss sealed-AM (PA2)	0.1502	0.446
S248	6 months sealed-AM (PA2)	0.0928	0.306
S249	6 months sealed-AL (PA2)	0.1295	0.406
S250	6 months sealed-AL (PA2)	0.096	0.582
S251	3 months exposed-AM (PA 1	0.0716	0.241
S252	3 months exposed-AM (PA 1	0.141	0.531
S253	3 months exposed-AL (PA 1)	0.1518	0.564
S254	3 months exposed-AL (PA 1)	0.1443	0.621
S255	6 months exposed-AM (PA 1)	0.137	0.712
S256	6 months exposed-AM (PA 1)	0.1118	0.66
S257	6 months exposed-AL (PA 1)	0.1222	0.677
S258	6 months exposed-AL (PA 1)	0.1137	0.602
S259	6M exposed-Abr+Wash-AM (PA 1)	0.0817	0.693
S260	6M exposed-Abr+Wash-AM(PA 1)	0.0701	0.602
S261	6M exposed-Abr+Wash-AL(PA 1)	0.051	0.79
S262	6M exposed-Abr+Wash-AL (PA1)	0.0707	0.584
S263	clay standard 1	0.1252	3.08
S264	clay standard 2	0.1369	3.42
S265	clay standard 3	0.1396	3.46

**Calibration curve 2**		**Area [µS*min]**	$C_{corrected}$ [ppm]	Mass Deviation [%]	$Mass_F$ [µg]
	2 ppm NaF	0.0114	1.79	90.05	0.18
	10 ppm NaF	0.0705	9.97	100.48	1.00
	20 ppm NaF	0.149	19.84	198.42	1.98
	25 ppm NaF	0.196	25.90	103.58	2.59
	50 ppm NaF	0.3779	48.45	96.90	4.84

Table S11: *TF concentrations for fabrics with C_4F_9-SFPs (based on calibration curves in Figure S14 a1)*

Sample	$C_{Fluorine}$ [µg]	C F [µg/g]	C F [wt%]	
stay control-AM (PA1)	4.376123235	5094.44	0.51	
stay control-AM (PA 1)	4.410783055	5128.82	0.51	
stay control-AL (PA 1)	3.086007702	4733.14	0.47	
stay control-AL (PA 1)	2.412066752	4033.56	0.40	outlier
shipping control-AM (PA 1)	4.314505777	5063.97	0.51	
shipping control-AM (PA1)	4.789473684	4897.21	0.49	
shipping control-AL (PA 1)	3.164313222	5309.25	0.53	
shipping control-AL (PA 1)	4.521181001	5440.65	0.54	
3 monthss sealed-AM (PA 2)	2.166880616	4542.73	0.45	
3 monthss sealedAM (PA 2)	1.901155327	4659.69	0.47	
3 monthss sealed-AL (PA 2)	1.585365854	4096.55	0.41	
3 monthss sealed-AL (PA 2)	1.811296534	2003.65	0.20	outlier
6 monthss sealed-AM (PA2)	2.026957638	4544.75	0.45	
6 months sealed-AM (PA2)	1.290115533	4216.06	0.42	
6 months sealed-AL (PA2)	1.761232349	4338.01	0.43	
6 months sealed-AL (PA2)	1.331193838	2287.27	0.23	outlier
3 months exposed-AM (PA 1	1.017971759	4223.95	0.42	
3 months exposed-AM (PA 1	1.90885751	3594.84	0.36	
3 months exposed-AL (PA 1)	2.047496791	3630.31	0.36	
3 months exposed-AL (PA 1)	1.951219512	3142.06	0.31	
6 months exposed-AM (PA 1)	1.857509628	2608.86	0.26	
6 months exposed-AM (PA 1)	1.534017972	2324.27	0.23	
6 months exposed-AL (PA 1)	1.667522465	2463.11	0.25	
6 months exposed-AL (PA 1)	1.558408216	2588.72	0.26	
6M exposed-Abr+Wash-AM (PA 1)	1.14762516	1656.02	0.17	
6M exposed-Abr+Wash-AM(PA 1)	0.998716303	1659.00	0.17	
6M exposed-Abr+Wash-AL(PA 1)	0.753530167	953.84	0.10	
6M exposed-Abr+Wash-AL (PA1)	1.006418485	1723.32	0.17	
clay standard 1	1.706033376	553.91	0.06	
clay standard 2	1.856225931	542.76	0.05	
clay standard 3	1.890885751	546.50	0.05	

Table S12: *Set of CIC measurements for fabrics with C_6F_{13}-SFPs*

Sample	Area [µS*min]	
boat blank 1	0.0006	before calibration
boat blank 2	0.0004	
boat blank 3	0.0004	
boat blank 4	0.0016	during measurements
boat blank 5	0.0013	
boat blank 6	0.0012	
boat blank 7	0.0018	
boat blank 8	0.0016	
boat blank 9	0.0011	
boat blank 10	0.0053	
boat blank 11	0.0018	after calibation 2

Note: 100µl=0.1g was dosed to the boats

**Calibration curve 1**		**Area [µS*min]**	$C_{corrected}$ [ppm]	Mass Deviation [%]	$Mass_F$ [µg]
	2 ppm NaF	0.0094	1.79	90.05	0.18
	10 ppm NaF	0.0576	9.97	100.48	1.00
	20 ppm NaF	0.1231	19.84	198.42	1.98
	25 ppm NaF	0.1689	25.90	103.58	2.59
	50 ppm NaF	0.3322	48.45	96.90	4.84

**Fabric mesurments**		**Area [µS*min]**	Weigt fabrics [mg]
S146	stay control-AM (PA6)	0.1652	0.522
S147	stay control-AM (PA 6)	0.1115	0.367
S148	stay control-AL (PA 6)	0.2529	0.815
S149	stay control-AL (PA 6)	0.1709	0.558
S150	shipping control-AM (PA 3)	0.2062	0.658
S151	shipping control-AM (PA 3)	0.2192	0.717
S152	shipping control-AL (PA 3)	0.2476	0.796
S153	shipping control-AL (PA 3)	0.178	0.583
S268	3 months sealed-AM (PA 5)	0.1786	0.601
S269	3 months sealedAM (PA 5)	0.224	0.755
S270	3 months sealed-AL (PA 5)	0.1389	0.452
S271	3 months sealed-AL (PA 5)	0.1745	0.559
S154	6 months sealed-AM (PA6)	0.2135	0.692
S155	6 months sealed-AM (PA6)	0.1686	0.534
S156	6 months sealed-AL (PA6)	0.2601	0.831
S157	6 months sealed-AL (PA6)	0.2392	0.74
S158	3 months exposed-AM (PA 5)	0.2164	0.711
S159	3 months exposed-AM (PA 5)	0.2627	0.866
S160	3 months exposed-AL (PA 5)	0.2462	0.796
S161	3 months exposed-AL (PA 5)	0.1823	0.523
S162	6 months exposed-AM (PA 6)	0.1942	0.649
S163	6 months exposed-AM (PA 6)	0.2207	0.785
S164	6 months exposed-AL (PA 6)	0.1742	0.577
S165	6 months exposed-AL (PA 6)	0.2348	0.781
S166	6M exposed-Abr+Wash-AM (PA 6)	0.0896	0.395
S167	6M exposed-Abr+Wash-AM(PA 6)	0.0925	0.424
S168	6M exposed-Abr+Wash-AL(PA 6)	0.099	0.399
S169	6M exposed-Abr+Wash-AL (PA6)	0.16	0.617
S170	clay standard 1	0.9107	23.39
S171	clay standard 2	0.7388	19.74
S172	clay standard 3	1.005	25.79

Calibration curve 2		**Area [µS*min]**	$C_{corrected}$ [ppm]	Mass Deviation [%]	$Mass_F$ [µg]
	2 ppm NaF	0.011	1.79	90.05	0.18
	10 ppm NaF	0.0613	9.97	100.48	1.00
	20 ppm NaF	0.1298	19.84	198.42	1.98
	25 ppm NaF	0.1714	25.90	103.58	2.59
	50 ppm NaF	0.3358	48.45	96.90	4.84

Table S13: *TF concentrations for fabrics with C_6F_{13}-SFPs (based on calibration curves in Figure S14 a2)*

Sample	$C_{Fluorine}$ [µg]	C F [µg/g]	C F [wt%]	
stay control-AM (PA6)	2.451428571	4696.22	0.47	
stay control-AM (PA 6)	1.684285714	4589.33	0.46	
stay control-AL (PA 6)	3.704285714	4545.14	0.45	
stay control-AL (PA 6)	2.532857143	4539.17	0.45	
shipping control-AM (PA 3)	3.037142857	4615.72	0.46	
shipping control-AM (PA 3)	3.222857143	4494.92	0.45	
shipping control-AL (PA 3)	3.628571429	4558.51	0.46	
shipping control-AL (PA 3)	2.634285714	4518.50	0.45	
3 months sealed-AM (PA 5)	2.642857143	4397.43	0.44	
3 months sealedAM (PA 5)	3.291428571	4359.51	0.44	
3 months sealed-AL (PA 5)	2.075714286	4592.29	0.46	
3 months sealed-AL (PA 5)	2.584285714	4623.05	0.46	
6 months sealed-AM (PA6)	3.141428571	4539.64	0.45	
6 months sealed-AM (PA6)	2.5	4681.65	0.47	
6 months sealed-AL (PA6)	3.807142857	4581.40	0.46	
6 months sealed-AL (PA6)	3.508571429	4741.31	0.47	
3 months exposed-AM (PA 5)	3.182857143	4476.59	0.45	
3 months exposed-AM (PA 5)	3.844285714	4439.13	0.44	
3 months exposed-AL (PA 5)	3.608571429	4533.38	0.45	
3 months exposed-AL (PA 5)	2.695714286	**5154.33**	0.52	outlier
6 months exposed-AM (PA 6)	2.865714286	4415.58	0.44	
6 months exposed-AM (PA 6)	3.244285714	4132.85	0.41	
6 months exposed-AL (PA 6)	2.58	4471.40	0.45	
6 months exposed-AL (PA 6)	3.445714286	4411.93	0.44	
6M exposed-Abr+Wash-AM (PA 6)	1.371428571	3471.97	0.35	
6M exposed-Abr+Wash-AM(PA 6)	1.412857143	3332.21	0.33	
6M exposed-Abr+Wash-AL(PA 6)	1.505714286	3773.72	0.38	
6M exposed-Abr+Wash-AL (PA6)	2.377142857	3852.74	0.39	
clay standard 1	13.10142857	560.13	0.06	
clay standard 2	10.64571429	539.30	0.05	
clay standard 3	14.44857143	560.24	0.06	

Table S14: *Set of CIC measurements for fabrics with C_8F_{17}-SFPs*

Calibration curve 1		Area [µS*min]	$C_{corrected}$ [ppm]	Mass Deviation [%]	$Mass_F$ [µg]
	2 ppm NaF	0.0102	1.79	90.05	0.18
	10 ppm NaF	0.0629	9.97	100.48	1.00
	20 ppm NaF	0.1376	19.84	198.42	1.98
	25 ppm NaF	0.1857	25.90	103.58	2.59
	50 ppm NaF	0.3641	48.45	96.90	4.84
**Fabric mesurments**		**Area [µS*min]**	Weigt fabrics [mg]		
S204	stay control-AM (PA8)	0.2268	0.70752		
S205	stay control-AM (PA8)	0.1645	0.451		
S206	stay control-AL (PA 8)	0.2389	0.7		
S207	stay control-AL (PA8)	0.2117	0.634		
S208	shipping control-AM (PA 8)	0.2075	0.588		
S209	shipping control-AM (PA 8)	0.2188	0.639		
S210	shipping control-AL (PA 8)	0.1488	0.434		
S211	shipping control-AL (PA 1)	0.319	0.94		
S212	3 months sealed-AM (PA 8)	0.2011	0.61		
S213	3 months sealedAM (PA 8)	0.1953	0.617		
S214	3 months sealed-AL (PA 8)	0.1311	0.421		
S215	3 months sealed-AL (PA 8)	0.183	0.47		
S216	6 months sealed-AM (PA8)	0.2458	0.752		
S217	6 months sealed-AM (PA8)	0.227	0.716		
S218	6 months sealed-AL (PA8)	0.1447	0.48		
S219	6 months sealed-AL (PA8)	0.1766	0.575		
S220	3 months exposed-AM (PA 8)	0.2177	0.781		
S221	3 months exposed-AM (PA 8)	0.1826	0.637		
S222	3 months exposed-AL (PA 8)	0.2007	0.696		
S223	3 months exposed-AL (PA 8)	0.1879	0.661		
S224	6 months exposed-AM (PA 8)	0.158	0.615		
S225	6 months exposed-AM (PA 8)	0.1564	0.612		
S226	6 months exposed-AL (PA 8)	0.1796	0.716		
S227	6 months exposed-AL (PA 8)	0.1711	0.69		
S228	6M exposed-Abr+Wash-AM (PA 8)	0.1156	0.788		
S229	6M exposed-Abr+Wash-AM(PA 8)	0.1014	0.674		
S230	6M exposed-Abr+Wash-AL(PA 8)	0.1798	0.856		
S231	6M exposed-Abr+Wash-AL (PA 8)	0.1717	0.811		
S232	clay standard 1	0.2032	5.28		
S233	clay standard 2	0.0651	1.8		
S234	clay standard 3	0.0909	2.39		
Calibration curve 2		**Area [µS*min]**	$C_{corrected}$ [ppm]	Mass Deviation [%]	$Mass_F$ [µg]
	2 ppm NaF	0.0111	1.79	90.05	0.18
	10 ppm NaF	0.0671	9.97	100.48	1.00
	20 ppm NaF	0.1433	19.84	198.42	1.98
	25 ppm NaF	0.192	25.90	103.58	2.59
	50 ppm NaF	0.3683	48.45	96.90	4.84

Table S15: *TF concentrations for fabrics with C_8F_{17}-SFPs (based on calibration curves in Figure S14 a3)*

Sample	$C_{Fluorine}$ [µg]	C F [µg/g]	C F [wt%]	
stay control-AM (PA8)	3.095424837	4375.04	0.44	
stay control-AM (PA8)	2.281045752	5057.75	0.51	
stay control-AL (PA 8)	3.253594771	4647.99	0.46	
stay control-AL (PA8)	2.898039216	4571.04	0.46	
shipping control-AM (PA 8)	2.843137255	4835.27	0.48	
shipping control-AM (PA 8)	2.990849673	4680.52	0.47	
shipping control-AL (PA 8)	2.075816993	4782.99	0.48	
shipping control-AL (PA 1)	4.300653595	4575.16	0.46	
3 months sealed-AM (PA 8)	2.759477124	4523.73	0.45	
3 months sealedAM (PA 8)	2.683660131	4349.53	0.43	
3 months sealed-AL (PA 8)	1.844444444	4381.10	0.44	
3 months sealed-AL (PA 8)	2.522875817	5367.82	0.54	outlier
6 months sealed-AM (PA8)	3.34379085	4446.53	0.44	
6 months sealed-AM (PA8)	3.098039216	4326.87	0.43	
6 months sealed-AL (PA8)	2.022222222	4212.96	0.42	
6 months sealed-AL (PA8)	2.439215686	4242.11	0.42	
3 months exposed-AM (PA 8)	2.976470588	3811.10	0.38	
3 months exposed-AM (PA 8)	2.517647059	3952.35	0.40	
3 months exposed-AL (PA 8)	2.754248366	3957.25	0.40	
3 months exposed-AL (PA 8)	2.586928105	3913.66	0.39	
6 months exposed-AM (PA 8)	2.196078431	3570.86	0.36	
6 months exposed-AM (PA 8)	2.175163399	3554.19	0.36	
6 months exposed-AL (PA 8)	2.478431373	3461.50	0.35	
6 months exposed-AL (PA 8)	2.367320261	3430.90	0.34	
6M exposed-Abr+Wash-AM (PA 8)	1.641830065	2083.54	0.21	
6M exposed-Abr+Wash-AM(PA 8)	1.45620915	2160.55	0.22	
6M exposed-Abr+Wash-AL(PA 8)	2.481045752	2898.42	0.29	
6M exposed-Abr+Wash-AL (PA 8)	2.375163399	2928.68	0.29	
clay standard 1	2.786928105	527.83	0.05	
clay standard 2	0.981699346	545.39	0.05	
clay standard 3	1.318954248	551.86	0.06	

Table S16: *Statistics for the CIC measurements for fabrics with C_4F_9-SFPs*

C4F9- SFP total fluorine concretions all [µg/g]

unexposed	unexposed send to AUS	3M sealed	6M sealed	3M exposed	6M exposed	6M exposed +ABR and Wash
5094.4	5064.0	4542.7	4544.7	4223.949206	2608.861837	1656.024763
5128.8	4897.2	4659.7	4216.1	3594.835235	2324.269654	1658.997181
4733.1	5309.3	4096.6	4338.0	3630.313459	2463.105561	953.8356543
4033.6	5440.7	2003.6	2287.3	3142.060406	2588.717966	1723.319324
no outlier		outlier	outlier			

Anova

ANOVA

Source of Variation	SS	df	MS	F	P-value	F crit
Between Groups	1463705.546	3	487901.8487	4.115269225	0.03842819	3.708264819
Within Groups	1185589.136	10	118558.9136			
Total	2649294.682	13				

p< is smaller than 0.05 the controlls are different

F-Test Two-Sample for Variances

H0= Variances are equal; if p< 0.05 the variances are not equal; so for p>0.05 they are equal

	unexposed	unexposed send to AUS	3M sealed	6M sealed	3M sealed	3M exposed	6M sealed	6M exposed
Mean	4747.5	5177.8	4433.0	4366.3	4433.0	3647.8	4366.3	2496.2
Variance	258563.8	59351.9	88313.6	27607.5	88313.6	196943.8	27607.5	17302.5
Observations	4	4	3	3	3	4	3	4
df	3	3	2	2	2	3	2	3
F	4.356454638	Comment	3.19890391	Comment	0.448420144	Comment	1.596	Comment
P(F<=f) one-tail	0.129	Equal Variances	0.238	Equal Variances	0.325	Equal Variances	0.337	Equal Variances
F Critical one-tail	9.277		19.000		0.052		9.552	

t-Test: Two-Sample Assuming Equal Variances

when P< 0.05 there is a significant difference between the means

	unexposed	unexposed send to AUS	3M sealed	6M sealed	3M sealed	3M exposed	6M sealed	6M exposed
Mean	4747.5	5177.8	4433.0	4366.3	4433.0	3647.8	4366.3	2496.2
Variance	258563.8	59351.9	88313.6	27607.5	88313.6	196943.8	27607.5	17302.5
Observations	4	4	3	3	3	4	3	4
Pooled Variance	158957.8		57960.5		153491.7		21424.5	
Hypothesized Mean Difference	0		0		0		0	
df	6		4		5		5	
t Stat	-1.526260573		0.339403386		2.624099608	Comment	16.72769914	Comment
P(T<=t) one-tail	0.089		0.376		0.023	difference	0.000007	difference
t Critical one-tail	1.943	Comment	2.132	Comment	2.015		2.015	
P(T<=t) two-tail	0.178	no difference	0.751	no difference	0.047		0.000	
t Critical two-tail	2.447		2.776		2.571		2.571	

***Table S17:** Statistics for the CIC measurements for fabrics with C_6F_{13}-SFPs*

C6F13- SFP total fluorine concretions all [µg/g]

unexposed	unexposed send to AUS	3M sealed	6M sealed	3M exposed	6M exposed	6M exposed +ABR and Wash
4696.2	4615.7	4397.4	4539.6	4476.6	4415.6	3472.0
4589.3	4494.9	4359.5	4681.6	4439.1	4132.8	3332.2
4545.1	4558.5	4592.3	4581.4	4533.4	4471.4	3773.7
4539.2	4518.5	4623.1	4741.3		4411.9	3852.7
Anova				5154.3 Outlier		

ANOVA Single Factor

Source of Variation	*SS*	*df*	*MS*	*F*	*P-value*	*F crit*
Between Groups	45114.147	3	15038.04894	1.745202759	0.211007389	3.49029482
Within Groups	103401.5	12	8616.791866			
Total	148515.65	15				

p< is smaller than 0.05 the controlls are different

F-Test Two-Sample for Variances

H0= Variances are equal; if p< 0.05 the variances are not equal; so for p>0.05 they are equal

	unexposed	unexposed send to AUS	3M sealed	6M sealed	3M sealed	3M exposed	6M sealed	6M exposed	unexposed send to AUS	3M exposed
Mean	4592.5	4546.9	4493.1	4636.0	4493.1	4483.0	4636.0	4357.9	4546.91128	4483.034172
Variance	5285.3	2793.1	17908.2	8480.5	17908.2	2252.0	8480.5	23259.3	2793.084302	2251.991943
Observations	4	4	4	4	4	3	4	4	4	3
df	3	3	3	3	3	2	3	3	3	2
F	1.892	Comment	2.112	Comment	7.952	Comment	0.365	Comment	1.240272777	Comment
P(F<=f) one-tail	0.307	Equal Variances	0.277	Equal Variances	0.114	Equal Variances	0.215	Equal Variances	0.475469841	Equal Variances
F Critical one-tail	9.277		9.277		19.164		0.108		19.16429213	

t-Test: Two-Sample Assuming Equal Variances

when P< 0.05 there is a significant difference between the means

	unexposed	unexposed send to AUS	3M sealed	6M sealed	3M sealed	3M exposed	6M sealed	6M exposed	unexposed send to AUS	3M exposed
Mean	4592.5	4546.9	4493.1	4636.0	4493.1	4483.0	4636.0	4357.9	4546.91128	4483.034172
Variance	5285.3	2793.1	17908.2	8480.5	17908.2	2252.0	8480.5	23259.3	2793.084302	2251.991943
Observations	4.00	4.00	4.00	4.00	4.00	3.00	4.00	4.00	4	3
Pooled Variance	4039.20		13194.38		11645.73		15869.91		2576.647358	
Hypothesized Mean Difference	0.00		0.00		0.00		0.00		0	
df	6.00		6.00		5.00		6.00		5	
t Stat	1.01		-1.76		0.12	Comment	3.12	Comment	1.647628736	Comment
P(T<=t) one-tail	0.17		0.06		0.45	no difference	0.010	difference	0.080172569	difference
t Critical one-tail	1.94	Comment	1.94	Comment	2.02		1.94		2.015048373	
P(T<=t) two-tail	0.35	no difference	0.13	no difference	0.91		0.02		0.160345137	
t Critical two-tail	2.45		2.45		2.57		2.45		2.570581836	

Table S18: *Statistics for the CIC measurements for fabrics with C_8F_{17}-SFPs*

C_8F_{17}- SFP total fluorine concretions all [µg/g]

unexposed	unexposed send to AUS	3M sealed	3M sealed	3M exposed	6M exposed
4375.0	4835.3	4523.7	4446.5	3811.1	3570.9
5057.8	4680.5	4349.5	4326.9	3952.4	3554.2
4648.0	4783.0	4381.1	4213.0	3957.3	3461.5
4571.0	4575.2	5367.8	4242.1	3913.7	3430.9
no outlier		Outlier			

Anova

ANOVA Single Factor

Source of Variation	*SS*	*df*	*F*	*P-value*
Between Groups	448836.2443	3	4.87825142	0.021443641
Within Groups	337361.2293	11		
Total	786197.4736	14		

p< 0.05, differces in controlls

F-Test Two-Sample for Variances

H0= Variances are equal; if p< 0.05 the variances are not equal; so for p>0.05 they are equal

	unexposed	unexposed send to AUS	3M sealed	6M sealed	3M sealed	3M exposed	6M sealed	6M exposed
Mean	4663.0	4718.5	4418.1	4307.1	4418.1	3908.6	4307.1	3504.4
Variance	82478.0	13260.5	8614.5	10972.2	8614.5	4604.2	10972.2	4713.0
Observations	4	4	3	4	3	4	4	4
df	3	3	2	3	2	3	3	3
F	6.220		0.785		1.871		2.328	
P(F<=f) one-tail	0.084		0.468		0.297		0.253	
F Critical one-tail	9.277		0.052		9.552		9.277	

t-Test: Two-Sample Assuming Equal Variances

when P< 0.05 there is a significant difference between the means

	unexposed	unexposed send to AUS	3M sealed	6M sealed	3M sealed	3M exposed	6M sealed	6M exposed
Mean	4663.0	4718.5	4418.1	4307.1	4418.1	3908.6	4307.1	3504.4
Variance	82478.0	13260.5	8614.5	10972.2	8614.5	4604.2	10972.2	4713.0
Observations	4	4	3	4	3	4	4	4
Pooled Variance	47869.28152		10029.10803		6208.32074		7842.581373	
Hypothesized Mean Difference	0		0		0		0	
df	6		5		5		6	
t Stat	-0.35892836		1.451254921		8.466917102		12.8194833	
P(T<=t) one-tail	0.365970571		0.10320804		0.000188728		6.92073E-06	
t Critical one-tail	1.943180281		2.015048373		2.015048373		1.943180281	
P(T<=t) two-tail	0.731941142		0.20641608		0.000377455		1.38415E-05	
t Critical two-tail	2.446911851		2.570581836		2.570581836		2.446911851	

Table S19. *Targeted analysis of PFAAs in untreated fabrics. Only PFAS with detections above LOD are shown (all concentrations are shown in in ng/g).*

untreated

Name	place	exposure time	side chain		PFPrA	PFBA	PFPeA	PFHxA	PFHpA	PFOA	PFNA	PFDA	PFUnDA	PFDoDA	PFTriDA	PFTeDA	PFPeDA	PFHxDA	PFHpDA	8:2FTUCA	PFBS	PFPeS	PFHxS
				**LOD**	**0.757**	**1.618**	**0.261**	**1.299**	**0.685**	**0.663**	**0.564**	**0.323**	**0.227**	**0.079**	**0.009**	**0.012**	**0.008**	**0.105**	**0.019**	**0.002**	**0.537**	**0.001**	**0.022**
S111	ACES	0	unexposed	**1**	<LOD	<LOD	<LOD	1.368	<LOD	<LOD	<LOD	<LOD	<LOD	<LOD	<LOD	<LOD	0.013	<LOD	<LOD	<LOD	<LOD	<LOD	0.128
				**2**	2.297	<LOD	<LOD	1.304	<LOD	<LOD	<LOD	<LOD	<LOD	<LOD	<LOD	<LOD	0.022	0.121	<LOD	<LOD	<LOD	<LOD	0.260
				**3**	1.613	<LOD	<LOD	<LOD	<LOD	<LOD	<LOD	<LOD	<LOD	<LOD	0.021	0.049	<LOD	0.128	<LOD	0.005	<LOD	<LOD	0.110
				**average**	1.955	<LOD	<LOD	1.336	<LOD	<LOD	<LOD	<LOD	<LOD	<LOD	0.021	0.049	0.018	0.125	<LOD	0.005	<LOD	<LOD	0.166
				**σ**	0.483	<LOD	<LOD	0.045	<LOD	<LOD	<LOD	<LOD	<LOD	<LOD	-	-	0.006	0.005	<LOD	-	<LOD	<LOD	0.082
S112	Australia	6	6 months	**1**	2.685	<LOD	0.287	3.372	1.026	2.054	1.411	1.510	1.078	1.255	0.937	0.942	0.511	0.831	<LOD	0.564	<LOD	<LOD	0.067
				**2**	1.427	<LOD	0.307	2.614	0.828	2.166	2.064	2.576	1.955	1.832	1.291	0.687	0.390	0.677	<LOD	0.330	<LOD	0.011	0.167
				**3**	1.788	<LOD	<LOD	2.879	1.056	2.560	1.713	2.326	1.757	1.806	1.647	1.279	0.461	0.391	<LOD	0.439	<LOD	<LOD	0.185
				**average**	1.967	<LOD	0.297	2.955	0.970	2.260	1.729	2.138	1.597	1.631	1.291	0.969	0.454	0.633	<LOD	0.444	<LOD	0.011	0.140
				**σ**	0.648	<LOD	0.014	0.385	0.124	0.266	0.327	0.557	0.460	0.326	0.355	0.297	0.061	0.223	<LOD	0.117	<LOD	-	0.064

Table S20. *Targeted analysis of PFAAs in fabrics with C_4F_9-SFPs. Only PFAS with detections above LOD are shown. All concentrations are in ng/g.*

C_4F_9-SFP

Name	place	exposure time	side chain		PFPrA	PFBA	PFPeA	PFHxA	PFHpA	PFOA	PFNA	PFDA	PFUnDA	PFDoDA	PFTriDA	PFTeDA	PFPeDA	PFHxDA	PFHpDA	8:2FTUCA	PFBS	PFPeS	PFHxS
				**LOD**	**0.757**	**1.618**	**0.261**	**1.299**	**0.685**	**0.663**	**0.564**	**0.323**	**0.227**	**0.079**	**0.009**	**0.012**	**0.008**	**0.105**	**0.019**	**0.002**	**0.537**	**0.001**	**0.022**
S115	ACES	unexposed	C4	1	5.568	70.575	0.716	<LOD	<LOD	<LOD	<LOD	<LOD	<LOD	0.107	0.121	0.032	0.042	<LOD	<LOD	<LOD	37.650	<LOD	0.251
			PA1	2	6.842	67.935	0.765	<LOD	<LOD	<LOD	<LOD	<LOD	1.605	0.164	0.032	0.093	0.056	0.131	<LOD	0.013	36.419	<LOD	0.167
				3	7.378	80.530	0.983	<LOD	<LOD	<LOD	<LOD	<LOD	<LOD	0.125	0.080	0.131	0.011	<LOD	<LOD	<LOD	35.877	<LOD	0.326
S116	ACES	unexposed	C4	1	4.942	40.351	0.503	<LOD	<LOD	<LOD	<LOD	<LOD	<LOD	0.214	0.177	0.277	0.091	0.157	<LOD	0.018	24.050	<LOD	0.180
			PA3	2	4.190	47.399	0.419	<LOD	<LOD	<LOD	<LOD	0.429	0.322	0.317	0.249	0.181	0.122	0.268	<LOD	<LOD	27.331	0.004	0.206
				3	6.397	61.345	0.490	<LOD	<LOD	0.682	<LOD	0.453	0.771	0.685	0.477	0.312	0.185	<LOD	<LOD	0.019	29.819	<LOD	0.194
S114	Australia	unexposed send to AUS	C4	1	5.276	71.754	0.852	1.544	<LOD	0.663	<LOD	0.551	0.367	0.353	0.216	0.302	0.124	0.314	<LOD	0.005	36.440	<LOD	0.262
			PA1	2	6.069	85.059	0.849	1.870	<LOD	<LOD	<LOD	0.438	0.322	0.261	0.127	0.160	0.033	0.130	<LOD	<LOD	43.415	<LOD	0.229
				3	7.503	99.580	1.036	2.318	<LOD	<LOD	<LOD	0.429	<LOD	0.179	0.046	0.193	0.046	<LOD	<LOD	<LOD	49.358	<LOD	0.174
		unexposed		**average**	6.018	69.392	0.735	1.911	<LOD	0.673	<LOD	0.460	0.677	0.267	0.170	0.187	0.079	0.200	<LOD	0.014	35.595	0.004	0.221
				**σ**	1.125	18.309	0.222	0.389	<LOD	0.014	<LOD	0.052	0.552	0.177	0.137	0.096	0.056	0.086	<LOD	0.007	7.836	-	0.052
S137	Australia	6 months covered	C4	1	13.506	58.683	0.483	<LOD	<LOD	1.101	<LOD	<LOD	<LOD	0.105	0.092	0.087	0.015	<LOD	<LOD	0.005	15.253	0.005	0.336
			PA1	2	14.212	44.468	<LOD	<LOD	<LOD	1.450	<LOD	<LOD	<LOD	0.134	0.144	0.092	0.046	<LOD	<LOD	<LOD	15.342	<LOD	0.266
				3	14.622	66.246	1.407	1.518	<LOD	1.994	<LOD	<LOD	0.234	0.203	0.139	0.100	0.038	<LOD	<LOD	<LOD	18.374	0.015	0.453
				**average**	14.114	56.466	0.945	1.518	<LOD	1.515	<LOD	<LOD	0.234	0.147	0.125	0.093	0.033	<LOD	<LOD	0.005	16.323	0.010	0.352
					0.565	11.057	0.653	-	<LOD	0.450	<LOD	<LOD	-	0.050	0.029	0.007	0.016	<LOD	<LOD	-	1.777	0.006	0.095
S117	Australia	3 months	C4	1	345.718	2529.646	131.419	4.513	1.234	1.403	0.886	1.227	0.910	0.819	0.714	0.479	0.271	0.425	<LOD	0.015	1373.970	0.035	0.188
			PA 1	2	318.422	2679.228	140.855	4.641	1.276	1.218	0.689	0.821	1.017	0.815	0.720	0.542	0.214	1.014	0.028	0.032	1409.723	0.087	0.212
				3	387.254	2792.540	134.709	4.538	1.134	1.278	0.808	0.972	0.944	0.808	0.494	0.475	0.157	0.360	0.019	<LOD	1360.430	0.049	0.177
S118	Australia		C4	1	199.727	1497.348	85.255	4.524	1.375	1.253	0.695	0.711	0.628	0.635	0.359	0.391	0.175	0.490	<LOD	0.025	1020.158	0.090	0.165
			PA 3	2	188.624	1542.195	89.288	4.591	1.440	1.051	<LOD	0.575	0.487	0.525	0.305	0.301	0.124	0.319	<LOD	0.033	1170.684	0.091	0.067
				3	170.465	1443.562	84.938	4.797	1.343	1.205	0.605	0.915	0.753	0.698	0.634	0.600	0.257	0.420	<LOD	0.019	1120.260	0.098	0.248
				**average**	268.368	2080.753	111.077	4.601	1.300	1.235	0.736	0.870	0.790	0.717	0.538	0.465	0.200	0.505	<LOD	0.025	1242.537	0.075	0.176
				**σ**	93.036	648.496	27.143	0.108	0.109	0.114	0.110	0.226	0.204	0.120	0.180	0.106	0.058	0.256	<LOD	0.008	160.432	0.026	0.061
S119	Australia	6 months	C4	1	108.285	1155.587	62.868	5.776	1.749	3.847	3.710	4.841	4.867	4.422	3.153	2.898	1.031	1.994	0.395	0.215	1285.030	0.022	0.122
			PA 1	2	131.650	1519.980	78.015	6.482	3.343	3.871	3.737	3.993	4.349	4.349	3.300	3.283	1.247	2.530	0.515	0.146	1348.511	0.017	0.056
				3	129.044	1341.765	67.168	5.357	2.241	4.030	4.242	4.525	4.701	4.559	3.474	3.387	1.213	2.101	0.670	0.240	1189.307	0.014	0.141
S120	Australia		C4	1	115.013	1244.998	68.951	5.877	1.649	3.266	3.480	5.263	5.493	5.201	3.408	2.744	1.119	2.302	0.482	0.146	1451.175	<LOD	0.131
			PA 3	2	102.661	1094.139	61.701	5.247	3.587	4.973	4.501	5.545	6.896	6.246	4.299	3.401	1.533	2.941	0.581	0.168	1360.739	0.011	0.134
				3	126.576	1197.554	64.417	4.275	2.034	3.375	3.223	4.587	5.761	5.061	3.531	2.691	1.176	1.951	0.358	0.195	1440.466	0.014	0.156
				**average**	118.872	1259.004	67.187	5.502	2.434	3.894	3.816	4.792	5.344	4.973	3.527	3.067	1.220	2.303	0.500	0.185	1345.871	0.016	0.123
				**σ**	11.966	152.844	5.946	0.744	0.829	0.608	0.476	0.555	0.920	0.713	0.401	0.327	0.171	0.379	0.116	0.038	98.468	0.004	0.035
	unexposed	**C4 TOP unexposed**	C4	**LOD**	**12.0**	**12.0**	**8.9**	**7.4**	**10.1**	**3.4**	**3.4**	**8.3**	**12.0**	**3.4**	**14.2**	**3.4**	**3.4**	**-**	**-**	**-**	**3.0**	**-**	**5.7**
				1	2143.419	125428.738	12.528	8.335	13.839	8.732	6.712	<LOD	<LOD	<LOD	<LOD	<LOD	<LOD	-	-	-	372.773	-	<LOD
				2	2271.193	124527.204	11.647	<LOD	<LOD	4.448	3.706	<LOD	<LOD	<LOD	<LOD	<LOD	<LOD	-	-	-	344.852	-	<LOD
				3	2092.624	130529.051	10.705	<LOD	<LOD	4.229	<LOD	<LOD	<LOD	<LOD	<LOD	<LOD	<LOD	-	-	-	344.012	-	<LOD
				**average**	2169.078	126828.331	12.088	8.335	13.839	5.803	5.209	<LOD	<LOD	<LOD	<LOD	<LOD	<LOD	-	-	-	353.879	-	<LOD
				**σ**	92.008	3236.462	0.622	-	-	2.539	2.125	<LOD	<LOD	<LOD	<LOD	<LOD	<LOD	-	-	-	16.368	-	<LOD

Table S21. *Targeted analysis of PFAAs in fabrics with C_6F_{13}-SFPs. Only PFAS with detections above LOD are shown. All concentrations are in ng/g.*

C_6F_{13}-SFP

Name	place	exposure time	side chain		PFPrA	PFBA	PFPeA	PFHxA	PFHpA	PFOA	PFNA	PFDA	PFUnDA	PFDoDA	PFTriDA	PFTeDA	PFPeDA	PFHxDA	PFHpDA	8:2FTUCA	PFBS	PFPeS	PFHxS
				**LOD**	**0.757**	**1.618**	**0.261**	**1.299**	**0.685**	**0.663**	**0.564**	**0.323**	**0.227**	**0.079**	**0.009**	**0.012**	**0.008**	**0.105**	**0.019**	**0.002**	**0.537**	**0.001**	**0.022**
S122	ACES	unexposed	C6	1	0.987	2.241	0.864	3.852	<LOD	<LOD	<LOD	<LOD	0.363	0.297	0.258	0.204	0.109	0.275	<LOD	0.004	<LOD	0.001	0.178
			PA 3	2	1.089	3.072	0.931	4.889	<LOD	<LOD	<LOD	0.495	0.532	0.319	0.268	0.257	0.149	0.283	<LOD	<LOD	<LOD	0.010	0.202
				3	<LOD	3.132	0.877	4.273	<LOD	<LOD	<LOD	0.552	0.502	0.536	0.408	0.426	0.174	0.666	<LOD	<LOD	<LOD	<LOD	0.118
S123	ACES	unexposed	C6	1	1.269	16.742	0.854	8.777	<LOD	0.918	0.842	0.996	1.085	0.927	0.799	0.638	0.372	0.869	<LOD	0.007	<LOD	0.001	0.075
			PA 3	2	<LOD	<LOD	0.874	5.044	<LOD	0.718	<LOD	0.814	0.854	0.504	0.487	0.406	0.292	0.614	<LOD	<LOD	<LOD	<LOD	0.129
				3	<LOD	<LOD	1.198	6.721	<LOD	<LOD	<LOD	0.547	0.497	0.449	0.473	0.394	0.210	0.249	0.053	0.029	<LOD	0.010	0.138
S121	Australia	unexposed send to AUS	C6	1	1.104	7.061	0.792	3.339	<LOD	<LOD	<LOD	<LOD	0.288	0.161	0.122	0.092	0.040	0.213	<LOD	<LOD	5.505	0.001	0.107
			PA 6	2	0.991	7.256	0.913	3.093	<LOD	<LOD	<LOD	<LOD	<LOD	0.122	0.117	0.041	0.044	<LOD	0.019	<LOD	7.254	0.001	0.178
				3	0.969	42.572	0.821	22.423	<LOD	0.672	<LOD	<LOD	0.769	0.422	0.103	0.072	0.053	0.180	<LOD	0.004	4.597	<LOD	0.126
		unexposed		**average**	1.068	11.725	0.903	6.935	<LOD	0.769	0.842	0.681	0.611	0.415	0.337	0.281	0.160	0.419	0.036	0.011	5.785	0.004	0.139
				**σ**	0.113	14.474	0.119	6.076	<LOD	0.131	<LOD	0.216	0.269	0.240	0.229	0.200	0.116	0.259	0.024	0.012	1.350	0.005	0.040
S129	Australia	6 months covered	C6	1	12.574	1.829	1.211	7.519	<LOD	<LOD	<LOD	<LOD	<LOD	<LOD	<LOD	0.063	<LOD	0.106	<LOD	<LOD	<LOD	<LOD	0.098
			PA 6	2	10.779	<LOD	0.880	6.357	<LOD	<LOD	<LOD	<LOD	<LOD	0.115	0.026	0.035	0.035	0.205	<LOD	<LOD	<LOD	0.002	0.163
				3	7.482	2.184	1.063	5.477	<LOD	<LOD	<LOD	<LOD	0.550	<LOD	0.020	<LOD	<LOD	0.112	<LOD	<LOD	0.958	<LOD	0.061
				**average**	10.279	2.006	1.051	6.451	<LOD	<LOD	<LOD	<LOD	0.550	0.115	0.023	0.049	0.035	0.141	<LOD	<LOD	0.958	0.002	0.107
				**σ**	2.582	0.251	0.166	1.025	<LOD	<LOD	<LOD	<LOD	-	-	0.004	0.019	-	0.055	<LOD	<LOD	-	-	0.051
S125	Australia	3 months	C6	1	14.811	50.671	143.447	850.598	318.087	4.875	2.384	3.103	1.816	1.997	1.105	1.374	0.466	0.811	0.195	0.096	1.714	0.028	0.160
			PA3	2	16.721	54.720	142.362	792.726	319.073	4.922	2.953	3.646	1.962	2.144	1.444	1.153	0.563	1.085	0.372	0.047	1.618	0.050	0.118
				3	16.492	50.449	127.985	767.463	301.321	4.381	2.180	2.880	1.745	1.512	0.989	0.716	0.321	0.568	0.081	0.059	1.789	0.037	0.205
S126	Australia		C6	1	15.056	41.506	129.335	752.646	285.253	6.097	3.478	4.270	2.643	2.458	1.784	1.537	0.605	1.107	<LOD	0.109	0.900	0.047	0.120
			PA6	2	19.176	48.630	129.202	728.343	293.113	4.018	2.219	2.375	1.585	1.508	0.892	1.191	0.324	0.385	<LOD	0.045	1.090	0.027	0.265
				3	17.018	41.269	128.839	781.908	301.735	5.583	3.246	3.816	2.237	2.099	1.457	1.237	0.498	1.009	0.151	0.093	1.214	0.064	0.096
				**average**	16.545	47.874	133.528	778.948	303.097	4.980	2.743	3.348	1.998	1.953	1.279	1.201	0.463	0.828	0.200	0.075	1.388	0.042	0.161
				**σ**	1.575	5.405	7.286	41.754	13.439	0.763	0.558	0.691	0.385	0.376	0.340	0.276	0.119	0.297	0.124	0.028	0.368	0.014	0.064
S127	Australia	6 months	C6	1	9.326	43.801	144.541	911.767	526.537	4.505	4.606	4.641	4.658	3.616	2.748	2.137	0.837	1.470	0.234	0.062	1.621	0.008	0.136
				2	10.665	36.661	143.294	871.270	385.913	4.122	3.621	4.276	3.897	3.757	2.400	2.169	0.960	1.755	0.442	0.071	1.132	0.011	0.075
				3	10.025	43.006	133.138	815.842	456.911	4.737	3.525	4.608	4.137	3.779	2.356	1.838	0.827	1.773	0.235	0.103	1.316	0.002	0.168
S128	Australia		C6	1	19.553	53.694	199.909	1009.467	528.577	5.827	5.800	7.361	8.588	8.610	2.474	0.566	0.088	<LOD	<LOD	0.093	1.000	0.008	0.044
				2	16.540	40.062	155.805	854.831	481.063	4.863	4.951	7.473	9.995	10.744	2.855	0.731	0.057	<LOD	<LOD	0.119	0.981	<LOD	0.080
				3	14.090	33.993	108.124	724.221	462.556	3.940	4.326	5.085	5.596	4.296	0.486	0.019	0.037	<LOD	<LOD	0.088	1.166	0.001	<LOD
				**average**	13.366	41.870	147.469	864.566	473.593	4.666	4.472	5.574	6.145	5.800	2.220	1.243	0.467	<LOD	<LOD	0.089	1.203	0.006	0.101
				**σ**	4.090	6.889	30.328	95.326	52.858	0.670	0.855	1.451	2.545	3.087	0.872	0.920	0.449	<LOD	<LOD	0.021	0.239	0.005	0.050
	unexposed	**C6 TOP unexposed**	C6	**LOD**	**12**	**12**	**8.9**	**7.4**	**10.1**	**3.4**	**3.4**	**8.3**	**12**	**3.4**	**14.2**	**3.4**	**3.4**	**-**	**-**	**-**	**3.0**	**-**	**5.7**
				1	-	1483.274	4166.475	1087.830	176.452	6.647	5.695	<LOD	<LOD	<LOD	<LOD	<LOD	<LOD	-	-	-	<LOD	-	<LOD
				2	-	1365.765	3682.206	869.245	174.432	8.252	8.020	<LOD	<LOD	<LOD	<LOD	<LOD	<LOD	-	-	-	<LOD	-	<LOD
				3	-	1843.322	5067.370	1369.062	199.991	8.130	8.394	<LOD	<LOD	<LOD	<LOD	<LOD	<LOD	-	-	-	<LOD	-	<LOD
				**average**		1564.120	4305.350	1108.712	183.625	7.676	7.370	<LOD	<LOD	<LOD	<LOD	<LOD	<LOD	-	-	-	<LOD	-	<LOD
				**σ**		248.832	702.947	250.562	14.210	0.893	1.462	<LOD	<LOD	<LOD	<LOD	<LOD	<LOD	-	-	-	<LOD	-	<LOD
							outlier																

Table S22. *Targeted analysis of PFAAs in fabrics with C_8F_{17}-SFPs. Only PFAS with detections above LOD are shown. All concentrations are in ng/g.*

C_8F_{17}-SFP

Name	place	exposure time	side chain		PFPrA	PFBA	PFPeA	PFHxA	PFHpA	PFOA	PFNA	PFDA	PFUnDA	PFDoDA	PFTriDA	PFTeDA	PFPeDA	PFHxDA	PFHpDA	8:2FTUCA	PFBS	PFPeS	PFHxS
				**LOD**	**0.757**	**1.618**	**0.261**	**1.299**	**0.685**	**0.663**	**0.564**	**0.323**	**0.227**	**0.079**	**0.009**	**0.012**	**0.008**	**0.105**	**0.019**	**0.002**	**0.537**	**0.001**	**0.022**
S131	ACES	unexposed	C8	1	1.098	3.689	0.666	7.053	1.545	17.009	0.866	8.330	0.827	3.012	0.327	0.222	<LOD	<LOD	<LOD	0.051	<LOD	<LOD	0.121
			PA 6	2	1.715	4.944	0.687	5.076	0.734	9.565	<LOD	3.040	<LOD	1.045	0.012	<LOD	<LOD	<LOD	<LOD	0.050	<LOD	<LOD	0.099
				3	1.597	2.058	0.551	4.974	<LOD	8.210	<LOD	3.309	0.313	1.569	0.538	0.844	0.116	0.377	<LOD	0.022	<LOD	<LOD	0.095
S132	ACES	unexposed	C8	1	2.024	1.759	0.722	5.431	<LOD	12.564	1.418	4.822	1.106	1.076	<LOD	<LOD	<LOD	<LOD	<LOD	0.186	<LOD	<LOD	0.064
			PA 8	2	1.617	<LOD	0.604	4.120	1.012	12.862	1.652	4.913	0.987	1.787	<LOD	<LOD	0.010	<LOD	<LOD	0.100	<LOD	0.003	0.162
				3	1.826	1.984	1.063	5.000	1.198	14.443	0.622	5.586	0.496	1.737	<LOD	<LOD	0.039	<LOD	<LOD	0.159	<LOD	<LOD	0.072
S130	Australia	unexposed send to AUS	C8	1	1.098	3.689	0.666	7.053	1.545	17.009	0.866	8.330	0.827	3.012	0.327	0.222	<LOD	<LOD	<LOD	0.051	<LOD	<LOD	0.121
			PA 6	2	1.715	4.944	0.687	5.076	0.734	9.565	<LOD	3.040	<LOD	1.045	0.012	<LOD	<LOD	<LOD	<LOD	0.050	<LOD	<LOD	0.099
				3	1.597	2.058	0.551	4.974	<LOD	8.210	<LOD	3.309	0.313	1.569	0.538	0.844	0.116	0.377	<LOD	0.022	<LOD	<LOD	0.095
	Australia	unexposed		**average**	1.59	3.14	0.69	5.42	1.13	12.16	1.08	4.96	0.70	1.76	0.29	0.53	0.07	0.38	<LOD	0.08	<LOD	0.00	0.10
				**σ**	0.31	1.35	0.15	0.99	0.37	3.49	0.43	2.12	0.32	0.77	0.24	0.36	0.05	0.00	<LOD	0.06	<LOD	-	0.03
S135	Australia	6 months covered	C8	1	6.485	1.754	0.753	5.268	1.374	12.701	0.760	5.237	1.030	3.332	0.697	0.546	0.107	<LOD	<LOD	0.073	<LOD	0.005	0.169
			PA 8	2	7.033	<LOD	0.856	5.666	1.039	12.869	<LOD	5.102	0.628	2.474	0.357	<LOD	<LOD	<LOD	<LOD	0.051	<LOD	<LOD	0.193
				3	7.724	<LOD	0.917	5.770	1.505	15.876	0.716	7.292	0.715	2.625	0.117	<LOD	<LOD	<LOD	<LOD	0.055	<LOD	<LOD	0.105
				**average**	7.08	1.75	0.84	5.57	1.31	13.82	0.74	5.88	0.79	2.81	0.39	0.55	0.11	<LOD	<LOD	0.06	<LOD	0.00	0.16
				**σ**	0.62	-	0.08	0.27	0.24	1.79	0.03	1.23	0.21	0.46	0.29	-	-	<LOD	<LOD	0.01	<LOD	-	0.05
S138	Australia	3 months	C8	1	36.165	6.112	8.069	34.537	54.351	130.305	123.533	120.645	116.229	110.827	97.124	75.870	42.269	32.973	13.759	0.556	1.916	0.121	0.194
				2	32.003	10.863	3.842	40.145	74.589	192.301	202.175	221.416	210.142	204.678	165.861	122.350	66.416	47.651	17.705	0.952	1.695	0.105	0.183
				3	27.303	8.564	8.368	41.652	77.939	190.006	195.106	201.681	191.926	183.475	159.154	120.239	66.801	50.494	20.319	0.698	3.096	0.113	0.346
S139	Australia		C8	1	39.566	9.701	12.396	44.603	69.454	165.166	149.769	142.326	136.679	128.080	113.309	94.156	49.870	39.065	15.667	0.694	1.867	0.128	0.175
				2	28.446	11.702	20.788	73.061	144.701	338.926	371.952	387.789	387.026	370.606	294.102	221.197	102.006	77.237	24.597	1.656	1.886	0.087	0.149
				3	36.471	14.907	15.703	75.184	159.839	351.116	332.016	338.223	319.900	325.545	276.540	205.305	95.199	70.907	24.677	2.403	2.012	0.071	0.487
				**average**	33.326	10.308	11.528	51.530	96.812	227.970	229.092	235.347	226.984	220.535	184.348	139.853	70.427	53.054	19.454	1.160	2.079	0.104	0.256
				**σ**	4.873	2.981	6.082	17.816	43.971	93.459	100.316	106.732	106.001	105.600	82.676	59.622	23.898	17.540	4.568	0.725	0.509	0.022	0.133
S133	Australia	6 months	C8	1	14.950	9.158	31.198	140.190	238.174	493.358	527.777	536.491	772.699	901.614	699.351	244.563	18.900	8.860	<LOD	11.284	<LOD	0.013	0.044
			PA 6	2	15.117	8.813	32.144	134.780	259.962	477.873	499.555	481.235	702.958	763.089	569.265	191.900	12.379	2.672	<LOD	9.904	0.553	0.003	0.031
				3	16.937	8.343	27.232	120.813	196.059	396.602	435.208	459.167	677.009	773.283	534.306	171.540	9.422	1.456	0.055	9.998	0.585	0.003	<LOD
S134	Australia			1	14.510	7.694	28.030	130.815	279.374	553.858	564.357	595.663	723.263	740.973	376.294	71.565	5.337	3.983	<LOD	8.371	1.143	0.022	0.041
			PA 8	2	14.603	7.736	29.121	113.418	247.994	575.417	673.377	639.836	750.620	952.156	193.755	18.280	2.415	7.102	<LOD	13.302	1.103	<LOD	0.052
				3	14.571	8.285	29.197	121.610	264.324	580.998	624.864	642.110	744.716	874.552	488.762	112.887	9.999	11.800	0.073	7.793	1.396	0.001	0.052
				**average**	15.11	8.34	29.49	126.94	247.65	513.02	554.19	559.08	728.54	834.28	476.96	135.12	9.74	5.98	0.06	10.11	0.96	0.01	0.04
				**σ**	0.92	0.58	1.87	10.01	28.94	71.21	86.19	79.13	34.75	86.65	174.21	83.32	5.73	3.97	0.01	2.00	0.37	0.01	0.01
	unexposed	**C8 TOP unexposed**	C8	**LOD**	**12.0**	**12.0**	**8.9**	**7.4**	**10.1**	**3.4**	**3.4**	**8.3**	**12.0**	**3.4**	**14.2**	**3.4**	**3.4**	**-**	**-**	**-**	**3.0**	**-**	**5.7**
				1	-	320.98	776.99	1241.12	2644.08	948.42	561.06	314.64	217.04	118.75	43.66	23.21	<LOD	-	-	-	<LOD	-	<LOD
				2	-	285.66	757.45	1180.63	2802.76	834.21	527.30	324.64	281.18	165.80	44.00	46.84	<LOD	-	-	-	<LOD	-	<LOD
				3	-	244.79	590.51	924.53	2126.90	725.97	380.54	243.07	134.51	63.41	<LOD	9.86	<LOD	-	-	-	<LOD	-	<LOD
				**average**		283.812	708.315	1115.427	2524.582	836.202	489.633	294.118	210.908	115.986	43.830	26.635	<LOD	-	-	-	<LOD	-	<LOD
				**σ**		38.127	102.491	168.069	353.420	111.237	95.976	44.492	73.527	51.253	0.241	18.727	<LOD	-	-	-	<LOD	-	<LOD

Table S23. *Concentration of FTOHs measured in extracts of unexposed fabrics with C_8F_{17}-SFPs and conversion to fluorine content*

	**C 8:2 FTOH [ng/mg]**	**C 10:2 FTOH [ng/mg]**
Extract 1	2.11	1.04
Extract 2	1.77	1.19
Extract 3	2.67	1.57
C [ng/g]	2181.73	1265.39
σ [ng/g]	457.02	274.97

Calculation steps	**8:2 FTOH**	**10:2 FTOH**
Cx (mol)	8	10
Fx (mol)	17	21
M (F in FTOH) [g/mol]	323.0	399.0
M (FTOH) [g/mol]	464.12	564.1
Mass (F) i FTOHs [ng/g]	1518.23	894.92
σ [µg/g]	318.03	194.47
Mass (F) i FTOHs [µg/g]	**1.5**	**0.9**
σ [µg/g]	**0.3**	**0.2**

Table S21: *Summary of fluorine content calculations*

**Samples**	**C_8F_{17} SFP**	**C_6F_{13} SFP**	**C_4F_9 SFP**
TF loss after 6 months (CIC measurements) [µg/g]	1158.6	234.5	2489.2
σ [µg/g]	305.7	178.8	255.6
∑ PFAAs after TOP of textile extracts (unexposed)[µg/g]	6.0	4.6	80.3
σ [µg/g]	0.3	0.5	2.0
∑ PFAAs after weathering (6 months) [µg/g]	3.0	1.0	1.8
σ [µg/g]	0.2	0.1	0.1
∑ PFAAs after weathering (3 months) [µg/g]	1.3	0.9	2.3
σ [µg/g]	0.2	0.2	0.420

References

(1) Schultes, L.; Vestergren, R.; Volkova, K.; Westberg, E.; Jacobson, T.; Benskin, J. P. Per-and Polyfluoroalkyl Substances and Fluorine Mass Balance in Cosmetic Products from the Swedish Market: Implications for Environmental Emissions and Human Exposure. Environmental Science: Processes & Impacts 2018, 20 (12), 1680–1690.

(2) Gebbink, W. A.; Glynn, A.; Berger, U. Temporal Changes (1997–2012) of Perfluoroalkyl Acids and Selected Precursors (Including Isomers) in Swedish Human Serum. Environmental Pollution 2015, 199, 166–173. https://doi.org/10.1016/j.envpol.2015.01.024.

(3) Houtz, E. F.; Sedlak, D. L. Oxidative Conversion as a Means of Detecting Precursors to Perfluoroalkyl Acids in Urban Runoff https://pubs.acs.org/doi/pdf/10.1021/es302274g (accessed 2021 -05 -11). https://doi.org/10.1021/es302274g.

(4) Team, S. C. C. How Hot Does Your Roof Get? - Roof Surface Temperatures https://sentry-roof.com/news/how-hot-does-your-commercial-roof-really-get-in-the-summer/ (accessed 2021 -02 -19).

(5) Gaffin, S. R.; Imhoff, M.; Rosenzweig, C.; Khanbilvardi, R.; Pasqualini, A.; Kong, A. Y. Y.; Grillo, D.; Freed, A.; Hillel, D.; Hartung, E. Bright Is the New Black—Multi-Year Performance of High-Albedo Roofs in an Urban Climate. Environ. Res. Lett. 2012, 7 (1), 014029. https://doi.org/10.1088/1748-9326/7/1/014029.

(6) American Association of Textile Chemists & Colorists. AATCC TM111:2015e2 WEATHER RESISTANCE OF TEXTILES: EXPOSURE TO DAYLIGHT AND WEATHER https://members.aatcc.org/store/tm111/522/ (accessed 2020 -10 -20).

(7) Schellenberger, S.; Jonsson, C.; Mellin, P.; Levenstam, O.; Liagkouridis, I.; Ribbenstedt, A.; Hanning, A.-C.; Schultes, L.; Plassmann, M. M.; Persson, C.; Cousins, I. T.; Benskin, J. P. Release of Side-Chain Fluorinated Polymer-Containing Microplastic Fibers from Functional Textiles during Washing and First Estimates of Perfluoroalkyl Acid Emissions. Environmental Science & Technology 2019. https://doi.org/10.1021/acs.est.9b04165.

(8) Sztajnowski, S.; Krucińska, I.; Sulak, K.; Puchalski, M.; Wrzosek, H.; Bilska, J. Effects of the Artificial Weathering of Biodegradable Spun-Bonded PLA Nonwovens in Respect to Their Application in Agriculture. Fibres and Textiles in Eastern Europe 2012, 96, 89–95.

(9) Iacopi, A. V.; White, J. R. Residual Stress, Aging, and Fatigue Fracture in Injection Molded Glassy Polymers I. Polystyrene. Journal of Applied Polymer Science 1987, 33 (2), 577–606. https://doi.org/10.1002/app.1987.070330223.

(10) Paterson, M. W. A.; White, J. R. Effect of Water Absorption on Residual Stresses in Injection-Moulded Nylon 6,6. J Mater Sci 1992, 27 (22), 6229–6240. https://doi.org/10.1007/BF01133776.

(11) Sadanaga, Y.; Matsumoto, J.; Kajii, Y. Photochemical Reactions in the Urban Air: Recent Understandings of Radical Chemistry. Journal of Photochemistry and Photobiology C: Photochemistry Reviews 2003, 4 (1), 85–104. https://doi.org/10.1016/S1389-5567(03)00006-6.

(12) Carlsson, D. J.; Wiles, D. M. The Photooxidative Degradation of Polypropylene. Part I. Photooxidation and Photoinitiation Processes. Journal of Macromolecular Science, Part C 1976, 14 (1), 65–106. https://doi.org/10.1080/15321797608076113.

(13) White, J. R. Polymer Ageing: Physics, Chemistry or Engineering? Time to Reflect. Comptes Rendus Chimie 2006, 9 (11), 1396–1408. https://doi.org/10.1016/j.crci.2006.07.008.

(14) Ellis, D. A.; Martin, J. W.; De Silva, A. O.; Mabury, S. A.; Hurley, M. D.; Sulbaek Andersen, M. P.; Wallington, T. J. Degradation of Fluorotelomer Alcohols: A Likely Atmospheric Source of Perfluorinated Carboxylic Acids. Environmental Science & Technology 2004, 38 (12), 3316–3321. https://doi.org/10.1021/es049860w.

(15) Washington, J. W.; Jenkins, T. M. Abiotic Hydrolysis of Fluorotelomer-Based Polymers as a Source of Perfluorocarboxylates at the Global Scale. Environmental Science & Technology 2015, 49 (24), 14129–14135. https://doi.org/10.1021/acs.est.5b03686.

(16) van der Veen, I.; Hanning, A.-C.; Stare, A.; Leonards, P. E. G.; de Boer, J.; Weiss, J. M. The Effect of Weathering on Per- and Polyfluoroalkyl Substances (PFASs) from Durable Water Repellent (DWR) Clothing. Chemosphere 2020, 249, 126100. https://doi.org/10.1016/j.chemosphere.2020.126100.

(17) Yousif, E.; Haddad, R. Photodegradation and Photostabilization of Polymers, Especially Polystyrene: Review. SpringerPlus 2013, 2 (1), 398. https://doi.org/10.1186/2193-1801-2-398.

(18) Gijsman, P.; Meijers, G.; Vitarelli, G. Comparison of the UV-Degradation Chemistry of Polypropylene, Polyethylene, Polyamide 6 and Polybutylene Terephthalate. Polymer Degradation and Stability 1999, 65 (3), 433–441. https://doi.org/10.1016/S0141-3910(99)00033-6.

(19) Bengt G. Rånby, J. F. R. Photodegradation, Photo-Oxidation, and Photostabilization of Polymers: Principles and Applications; 1990.

(20) Gewert, B.; Plassmann, M. M.; MacLeod, M. Pathways for Degradation of Plastic Polymers Floating in the Marine Environment. Environmental Science: Processes & Impacts 2015, 17 (9), 1513–1521. https://doi.org/10.1039/C5EM00207A.

.
